# Dietary Tryptophan-Mediated Aryl Hydrocarbon Receptor Activation by the Gut Microbiota Alleviates Escherichia coli-Induced Endometritis in Mice

**DOI:** 10.1128/spectrum.00811-22

**Published:** 2022-06-21

**Authors:** Caijun Zhao, Lijuan Bao, Min Qiu, Lianjun Feng, Luotong Chen, Zhuoyu Liu, Shiyu Duan, Yihong Zhao, Keyi Wu, Naisheng Zhang, Xiaoyu Hu, Yunhe Fu

**Affiliations:** a Department of Clinical Veterinary Medicine, College of Veterinary Medicine, Jilin University, Changchun, Jilin Province, China; State Key Laboratory of Microbial Resources, Institute of Microbiology, Chinese Academy of Sciences

**Keywords:** gut microbiota, AhR, tryptophan metabolism, endometritis, *E. coli*, *Lactobacillus reuteri*

## Abstract

Intestinal microbiota-mediated aryl hydrocarbon receptor (AhR) activation plays an important role in host–microbiota interactions and disease development. However, whether AhR activation mediates infection-induced inflammation in remote organs is not clear. The purpose of this study is to assess the effects and underlying mechanism of AhR activation and gut microbiota-mediated dietary tryptophan (Trp) metabolism on infection-induced inflammation using an Escherichia coli (E. coli)-induced endometritis model in mice. We found that AhR activation by 6-formylindolo (3,2-b) carbazole (Ficz), which is an AhR agonist derived from the photooxidation of Trp, alleviated E. coli-induced endometritis by repairing barrier function and inhibiting inflammatory responses, while inhibition of AhR by CH223191, which is a synthetic AhR antagonist, aggravated E. coli-induced endometritis. Gut dysbiosis damaged AhR activation and exacerbated E. coli-induced endometritis in mice, which responded to the reduced abundance of AhR ligand producers, such as *Lactobacillus* spp. Supplementation with dietary Trp ameliorated E. coli-induced endometritis in a microbiota-dependent manner, which was associated with the production of AhR ligands. Administration of AhR ligands, including indole and indole aldehyde, but not indole-3-propionic acid, rescued the protective effect of Trp on E. coli-induced endometritis in dysbiotic mice. Moreover, consumption of Lactobacillus reuteri (L. reuteri) containing AhR ligand-producing capability also alleviated E. coli-induced endometritis in mice in an AhR-dependent manner. Our results demonstrate that microbiota-mediated AhR activation is a key factor in fighting pathogen-caused inflammation, which leads to a potential strategy to regulate the gut microbiota and metabolism by dietary Trp or probiotics for the intervention of infectious diseases and reproductive health.

**IMPORTANCE** Infection-induced endometritis is a common and frequently occurring disease in humans and animals. Accumulating evidence suggests an important role of the gut microbiota in the development of infection-induced inflammation. Whether and how gut microbiota-mediated AhR activation regulates the pathogenesis of pathogen-induced endometritis remains unknown. The current study found that AhR activation ameliorated E. coli-induced endometritis, and inhibition of AhR produced negative results. Gut dysbiosis reduced the abundance of AhR ligand producers including *Lactobacillus* spp., damaged AhR activation, and exacerbated E. coli-induced endometritis. Supplementation with dietary Trp, AhR ligands, and L. reuteri containing AhR ligand-producing capability alleviated E. coli-induced endometritis in mice. Our results suggest an important role of microbiota-mediated AhR activation in the pathogenesis of endometritis and provide potential strategies for the intervention of infectious diseases and reproductive health by regulating the gut microbiota and metabolism.

## INTRODUCTION

The complex and orchestral collection of bacteria and other microorganisms, known as the gut microbiota, has been at the forefront of the interest in regulating host homeostasis and controlling diseases (1–3). These commensal communities develop a close relationship with the host, and changes in their composition and function are associated with host health and disease development (4, 5). Emerging evidence has increasingly unveiled the role of the gut microbiota in numerous diseases, including metabolic disease (6), autoimmune disease (7), cancer (8), and infectious diseases (9). Gut dysbiosis is connected to pathogen invasions in the gastrointestinal tract and distant organs. For example, patients with Clostridium difficile and Vibrio cholerae colonization in the intestine have dysfunction of the gut microbiota (10–12). Similarly, gut dysbiosis individuals evolved increased susceptibility to viral infections, such as human immunodeficiency virus and norovirus (13, 14). In addition, there is an emerging association between the gut microbiota and female reproductive diseases, such as endometritis (15), which is commonly caused by opportunistic pathogens, such as Escherichia coli (E. coli) (16), and exhibits classical inflammation symptoms including redness, swelling, heat, and pain, which cause increased health risks for women and large economic losses for animals. A previous study implied pathogen transmission between the gut and uterus through blood (17). Our previous study also demonstrated that gut dysbiosis contributed to the pathogenesis of endometritis in mice (18). However, the specific role of the gut microbiota in endometritis needs more evidence, and the potential microbial mechanism underlying the gut–uterus connection is not clear.

Among the typical mechanisms of microbe–host interactions, most essential and effective is gut microbial metabolism, in which the gut microbiota produces numerous metabolites and small reactive molecules, which reach and accumulate in the gut and distant organs and affect host physiology and disease pathogenesis (19). A good understanding of gut microbial metabolism is exemplified by aromatic amino acid metabolism, short-chain fatty acid metabolism, and bile acid metabolism (20–23). Tryptophan (Trp)-derived aryl hydrocarbon receptor (AhR) ligands are included in the array of microbiota-associated metabolites connecting host–microbiota intersections and activate AhR, which is a basic transcription factor expressed by most cell types and regulates cellular responses to milieu stimuli including commensal microbes (24, 25). AhR is a ligand-activated transcription factor expressed by a number of immune cells, and it is activated by small molecules provided primarily by gut microbial metabolism (26, 27). AhR signaling provides a complex molecular pathway that integrates the effects of the microbiota and metabolism on the immune response (26, 27), which leads to its essential roles in the immune system and mediates the outcomes of diseases (26). Dysfunction of AhR activation is associated with the deterioration of diseases. A prior study indicated that caspase recruitment domain family member 9 knockout (CARD9^−/−^) mice had increased susceptibility to colitis, causing altered gut microbiome and damaged Trp metabolism (28), while AhR activation by supplementation with 6-formylindolo (3,2-b) carbazole (Ficz), a typical Trp-derived AhR agonist (29), alleviated colitis scores in CARD9^−/−^ mice (28). Reduced AhR ligand production in feces was detected in mice with high-fat diet (HFD) consumption-induced metabolic syndrome, and activation of AhR by Ficz ameliorated HFD-caused metabolic impairments (6). These studies suggest that restoration of AhR signaling contributes to disease recovery. Notably, inflammatory stimuli affect Trp metabolism and AhR activation, which negatively regulate infection or inflammatory injury and maintain host mucosal homeostasis. Alvarado et al. found that enteropathogenic E. coli or inflammatory stimuli, such as dextran sodium sulfate, activated AhR via indoleamine 2,3-dioxygenase 1 (IDO1), which increased the resistance to enteropathogenic E. coli infection and colitis (30). Another study indicated that macrophages expressed and produced interleukin (IL)-22 after activation of AhR when cells were activated by the Toll-like receptor (TLR) family (31). The main virulence factor of E. coli, lipopolysaccharide (LPS), directly activated AhR, which in turn limits the LPS-induced inflammatory response in macrophages (32). AhR participates in the physiological activity of the uterus (33), and E. coli is one of the main pathogens of endometritis (34, 35), which indicates that the AhR signaling pathway may play an important role in the pathogenesis of E. coli-induced endometritis.

Commensal microorganisms contain Trp metabolism-related enzymes that are responsible for AhR ligand production, including Lactobacillus reuteri (L. reuteri), Arthrobacter pascens, and Clostridium sporogenes (36, 37). Supplementation with bacteria with AhR ligand-producing capability may be a potential strategy for disease interventions. The well characterized is L. reuteri, with great capacity for AhR ligand production, although *Lactobacilli* have been involved in the modulation of gut homeostasis and host immunity in multiple manners (38). Supplementation with L. reuteri decreased colon inflammation in CARD9^−/−^ mice, alleviated HFD-induced metabolic syndrome, and limited the colonization of pathogens (6, 28, 39). The primary mechanism of AhR activation or L. reuteri administration is the production of IL-22 derived from type 3 innate lymphoid cells (ILC3s) and direct inhibition of the nuclear factor kappa beta (NF-κB) pathway, leading to enhanced barrier integrity and limited pathogen-related inflammation (6, 28, 37). Notably, *Lactobacillus* species are the predominant bacterial taxa in the healthy gut microenvironment (40, 41), which suggests potential protective roles of *Lactobacillus* and AhR in host homeostasis. Collectively, AhR activation participates in many diseases; however, it is unclear whether and how AhR activation regulates the development of infection-induced inflammation in remote organs and the effects of dietary Trp and commensal probiotic supplementation.

Given that the known gut microbiota is associated with endometritis pathogenesis and the key role of AhR in host–microbiota intersections, especially in mucosal protection, we hypothesized that damaged AhR activation by reduced microbiota-related AhR ligand production plays an important role in endometritis. The present study demonstrated that activating AhR alleviated *E.coli*-induced endometritis in mice. Gut dysbiosis impaired AhR activation, which resulted in an *E.coli*-induced increase in endometritis score. Supplementation with an AhR agonist, dietary Trp and its metabolites, and a *Lactobacillus* strain with high AhR ligand-producing capacity, to compensate for the damaged microbiota-derived AhR ligand signaling, alleviated E. coli*-*induced endometritis. The underlying mechanism is involved in the improvement of barrier function and inflammation restriction.

## RESULTS

### AhR activation ameliorates E. coli-induced endometritis in mice.

**FIG 1:**
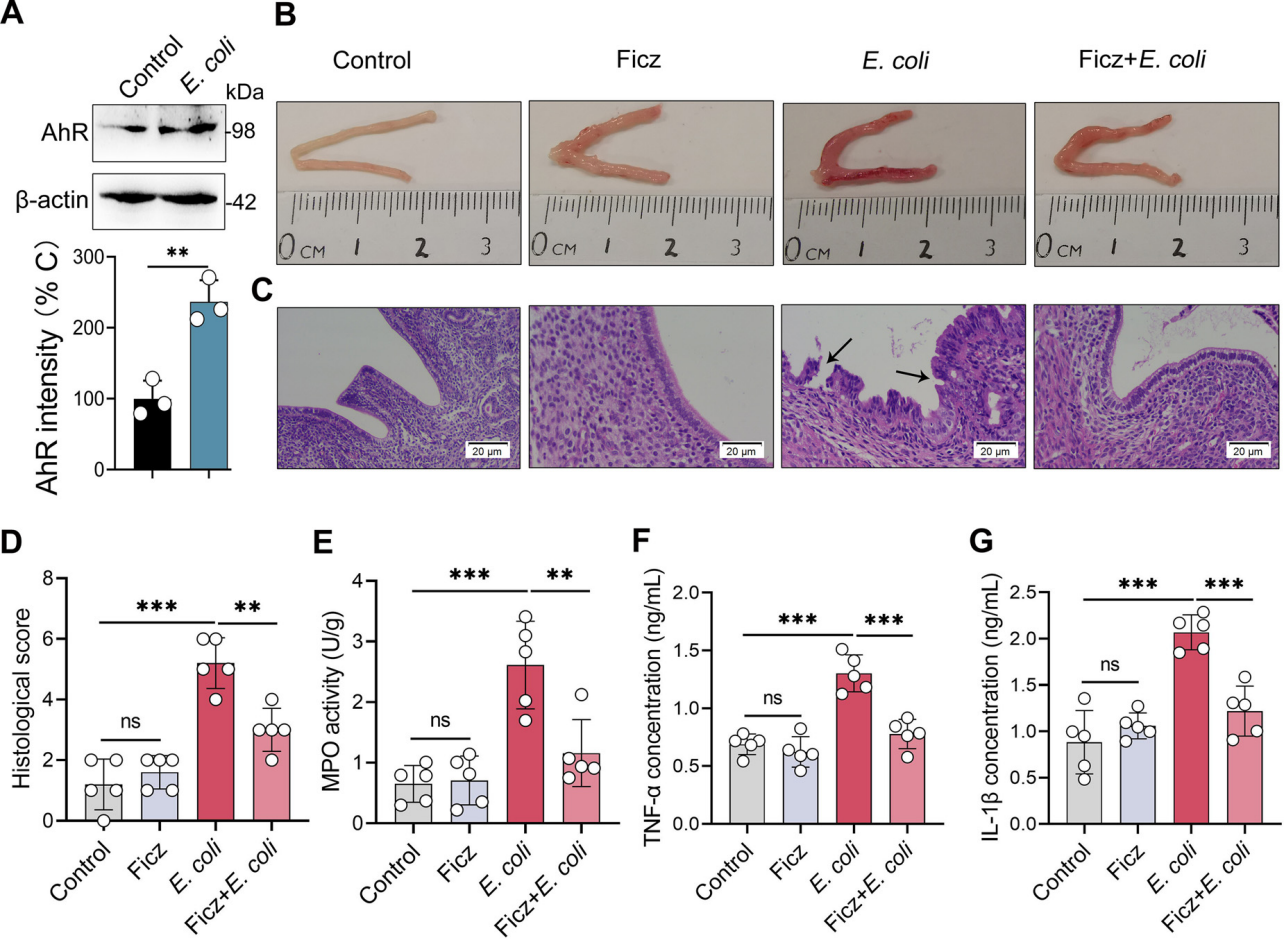
AhR activation improves the immunopathology of E. coli-induced endometritis in mice. (A) Uterine AhR expression was assessed in control and E. coli-induced endometritis mice. Mice were treated with E. coli (10^8^ CFU/30 μL on each side of uterus) by intrauterine injection. After 24 h, uterine tissues were harvested and determined by Western blotting (*n* = 3). (B-F) Mice were pretreated with Ficz (50 μg/kg BW) intraperitoneally 1 h before E. coli administration (10^8^ CFU/30 μL on each side of uterus). The control group was treated with equal DMSO intraperitoneally and PBS in the uterus. (B-C) Representative macroscopic images (B) and H&E-stained images (C) from different treated mice are shown. E. coli-treated group had increased macroscopic inflammatory changes compared with other groups, including redness and edema. The black arrow indicates endometrial injury (scale bar = 20 μm). (D) Histological scores in different treatment groups were performed (*n* = 5). (E-G) MPO activity (E), TNF-α (F), and IL-1β (G) levels from the indicated mice were determined (*n* = 5). Data are expressed as the mean ± SD. Two-tailed Student's *t* test (A) and one-way analysis of variance (ANOVA; D-G) were performed. *, *P < *0.05; **, *P < *0.01; and ***, *P < *0.001 indicate significant differences. ns, no significance.

To investigate whether AhR activation was associated with E. coli-induced endometritis, we measured the protein expression of AhR in uterine tissues from control and *E.coli*-induced endometritis mice. Notably, higher AhR level was detected in the *E.coli* treatment group than the control group (Fig. 1A), which suggests that the AhR pathway participates in the pathogenesis of E. coli-induced endometritis. To study the effects of AhR activation on endometritis, we pretreated mice with Ficz, an AhR agonist (6, 28, 42), followed by E. coli stimulation. E. coli-infected mice developed obvious inflammatory responses, as shown by macroscopic redness and edema (Fig. 1B), endometrial barrier function disruption (Fig. 1C), and several markers of inflammation (Fig. 1C–G). Ficz treatment reversed E. coli*-*induced inflammation and barrier injury, which were characterized by improved macroscopic changes (Fig. 1B), lower levels of hyperemia, inflammatory cell infiltration, and epithelial barrier lesions than E. coli treatment (Fig. 1C and 1D), and reduced myeloperoxidase (MPO) activity (Fig. 1E), TNF-α (Fig. 1F), and IL-1β (Fig. 1G) levels compared with the E. coli group.

**FIG 2:**
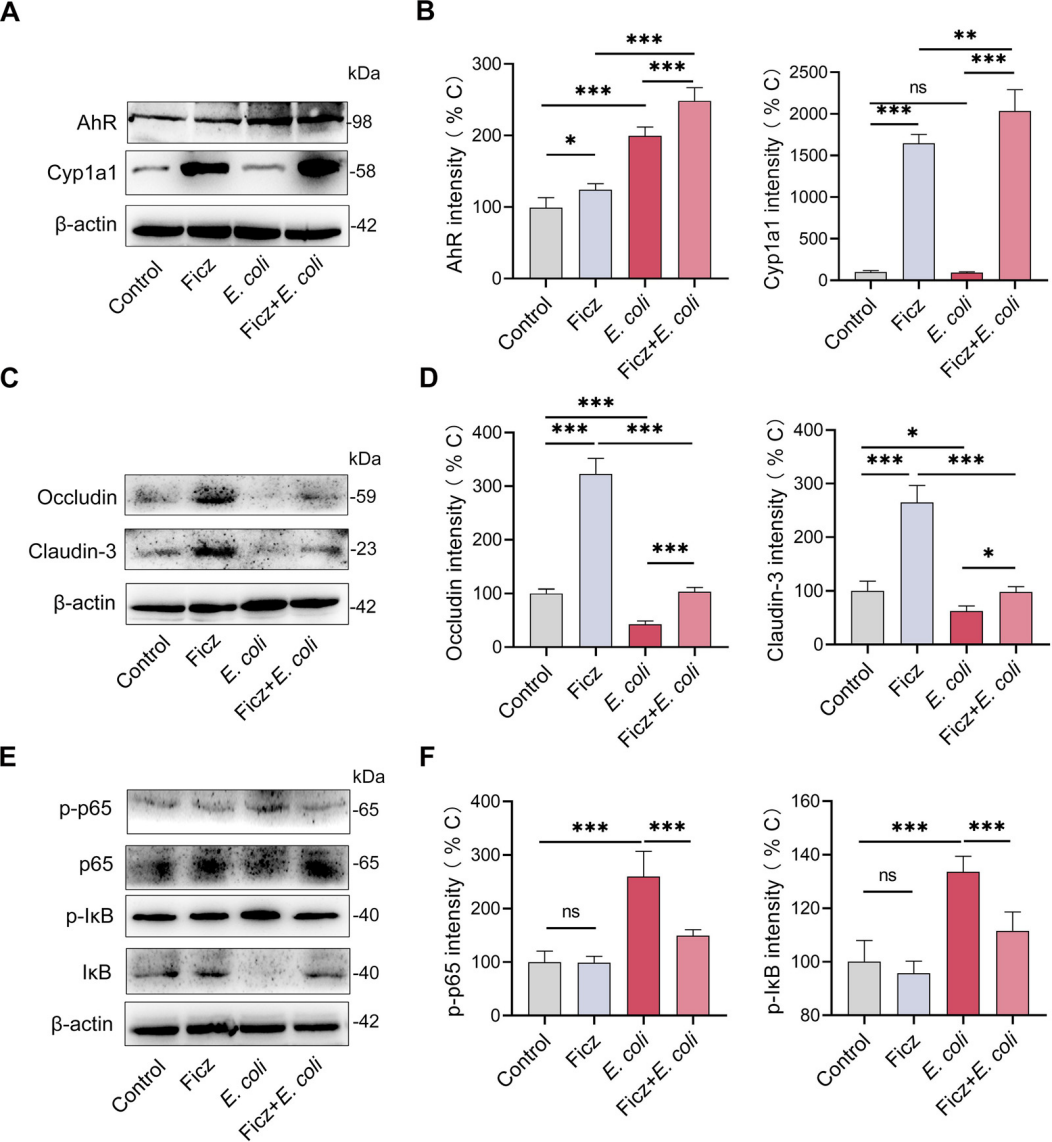
AhR activation increases tight junction protein levels and limits NF-κB activation. The mice were treated with Ficz and E. coli as mentioned above. (A) AhR and Cyp1a1 levels were assessed by Western blotting. (B) Intensity analysis of AhR and Cyp1a1 was performed based on Western blotting (*n* = 5). (C-D) Uterine occludin and claudin-3 protein levels (C) from indicated mice were determined by Western blotting, and intensity analysis of occludin and claudin-3 (D) was performed based on Western blotting (*n* = 5). (E-F) Representative images of p65, IκB, p-p65, and p-IκB from different treated groups. (E) Intensity analysis of p-p65 and p-IκB (F) were performed (*n* = 5). Data are expressed as the mean ± SD, and one-way ANOVA was performed (B, D, and F). *, *P < *0.05; **, *P < *0.01; and ***, *P < *0.001 indicate significant differences. ns, no significance.

To confirm the role of AhR activation in the Ficz-mediated protective effects on E. coli-induced endometritis, we detected the activation of the uterine AhR pathway by Western blotting. The results showed that Ficz increased AhR levels with or without E. coli stimulation (Fig. 2A and B). The AhR pathway is activated by direct ligand binding (37, 43) and inflammatory factors such as LPS (44), which induce different downstream signaling activation and modulate the development of various diseases (24, 25, 27). Therefore, we detected Cytochrome P450 1A1 (Cyp1a1) expression, which is responsible for AhR ligand depletion and prevents the overactivity of AhR (24, 25, 27). Indeed, Ficz treatment increased Cyp1a1 levels in uterine tissues compared with control mice, while E. coli stimulation did not affect Cyp1a1 levels (Fig. 2A and B). AhR activation accounts for mucosal inflammation limitation and barrier function maintenance (24, 25, 28), which are associated with the immunopathology of endometritis (15). We therefore investigated the effect of Ficz treatment on tight junction (TJ) proteins including occludin and claudin-3, and on activation of the NF-κB pathway, which is the main inflammatory transcriptional signaling pathway associated with E. coli-induced inflammatory responses (45, 46). Similar to previous studies (24, 47), Ficz treatment increased the levels of occludin and cladin-3 in uterine tissues compared with control mice, and reversed E. coli-induced decreases in occludin and claudin-3 (Fig. 2C and D). Higher levels of phosphorylation (p-) p65 and p-IκB were observed in *E.coli*-treated mice than control mice (Fig. 2E and F), which indicated activation of the NF-κB pathway (48). Notably, Ficz treated mice had lower levels of p-p65 and p-IκB than E. coli-treated mice (Fig. 2E and F), suggesting that AhR signaling inhibits activation of the NF-κB pathway. Collectively, these results suggest that AhR activation alleviates E. coli-induced endometritis by affecting barrier function and inflammatory signal transduction.

### Inhibition of the AhR pathway aggravates E. coli-induced endometritis in mice.

To further confirm whether the AhR pathway was required for host defense against E. coli-induced endometritis, we blocked AhR activation by pretreating mice with the specific AhR inhibitor CH223191 before Ficz and E. coli administration (49, 50). CH223191 pretreated mice had aggravated endometritis compared with E. coli treatment, as shown by increased macroscopic inflammatory changes (Fig. S1A in the supplemental material), barrier disruption and inflammatory infiltrations (Fig. S1B), histological score (Fig. S1C), MPO activity (Fig. S1D), inflammatory cytokines (Fig. S1E and F), and reduced occludin and claudin-3 levels (Fig. S1G–I) compared with E. coli-treated mice. Consistently, pretreatment with CH223191 also weakened the protective effects of Ficz on inflammation regulation and barrier repair in the uterus (Fig. S1A–I), which suggests that activation of the AhR signaling pathway is required for protecting against E. coli-caused uterine injury and inflammation.

### Gut dysbiosis impairs uterine AhR activation, reduces intestinal AhR producer abundance, and aggravates E. coli-induced endometritis in mice.

**FIG 3:**
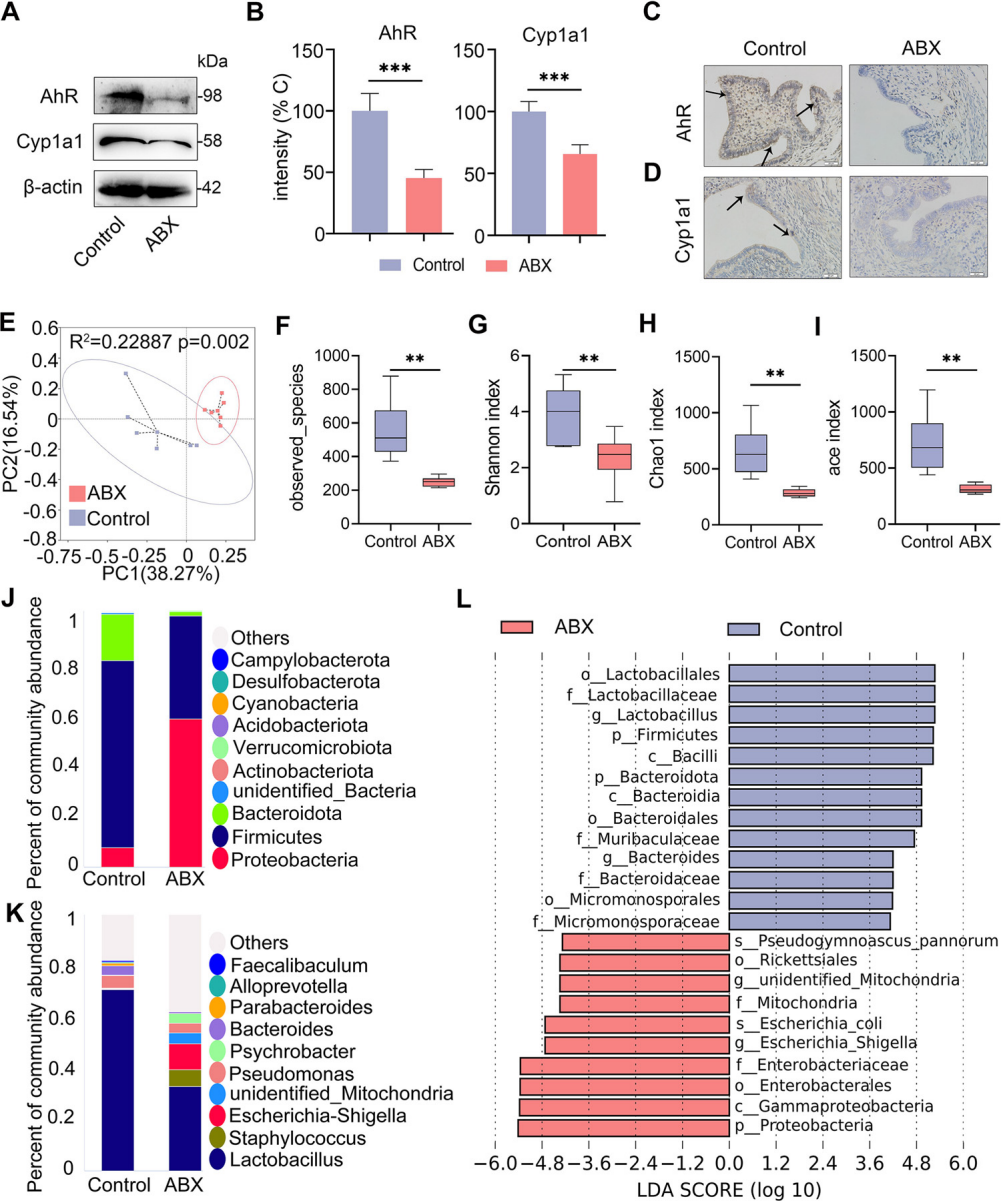
Gut dysbiosis impairs uterine AhR activation and reduces the abundance of intestinal AhR producers in mice. The mice were treated with ABX (1 g/L ampicillin, metronidazole, and neomycin sulfate and 0.5 g/L vancomycin) for 3 weeks, and fecal and uterine samples were harvested for determination. (A) Levels of uterine AhR and Cyp1a1 in control and ABX-treated mice were assessed by Western blotting. (B) Intensities of AhR and Cyp1a1 in the indicated mice were determined (*n* = 5). (C) Representative images of AhR antibody-stained uterine sections from control and ABX treated mice are shown. The arrow indicates positively stained cells (scale bar = 20 μm). (D) Representative images of Cyp1a1 antibody-stained sections are shown. The arrow indicates positively stained cells (scale bar = 20 μm). (E) Principal coordinates analysis (R^2^ = 0.22887, *P* = 0.002) shows distinct gut microbial structure between the control and ABX treatment groups based on unweighted UniFrac distances (*n* = 6). (F-I) Alpha diversity analyses, including observed species (F), Shannon (G), Chao1 (H), and ace index (I), were performed from different treatment groups (*n* = 6). (J-K) Gut bacterial compositions at the phylum (J) and genus (K) levels are shown. (L) Linear discriminant analysis (LDA) effect size (LEfSe) was performed to show the most differentially significant bacterial taxa enriched in the control and ABX groups (log_10_ LDA score > 4). Data are expressed as the mean ± SD (B) or boxplot (F-I), and two-tailed Student's *t* test (B) and Mann-Whitney *U* test were performed for statistical analysis (F-I). **, *P < *0.01; and ***, *P < *0.001 indicate significant differences. ABX, cocktail of antibiotics.

The gut microbiota participates in the development of endometritis in mice (18). To examine whether AhR was involved in gut microbiota-mediated protective effects against E. coli-induced endometritis, mice were treated with a cocktail of antibiotics (ABX) consisting of 1 g/L metronidazole, ampicillin and neomycin sulfate, and 0.5 g/L vancomycin for 3 weeks to disrupt the gut microbiota (45). We found that ABX-treated mice had lower uterine AhR and Cyp1a1 protein expression than control mice (Fig. 3A and B). To confirm these results, we determined the tissue localization of AhR and Cyp1a1 using immunohistochemistry. Consistently, we found that the ABX-treated group had reduced positive staining of AhR and Cyp1a1 (Fig. 3C and D). These results indicate that gut dysbiosis impairs AhR activation in the uterus. To verify the gut microbiota changes, we performed 16S rRNA sequencing in fecal contents. Principal coordinates analysis (PCoA) showed that ABX-treated mice had separated microbial structures from control mice based on unweighted UniFrac distances (Fig. 3E). Alpha diversity analysis, including observed species (Fig. 3F), Shannon (Fig. 3G), Chao1 (Fig. 3H) and ace (Fig. 3I) indices, showed that ABX-treated mice had reduced gut microbial diversity and richness compared with control mice. At the phylum level, ABX treatment reduced the abundance of *Fimicutes* and *Bacteroidota* but increased the *Proteobacteria* abundance compared with the control group (Fig. 3J). At the genus level, the ABX treatment group also had distinct microbial compositions compared with the control group, especially *Lactobacillus* (Fig. 3K). Linear discriminant analysis (LDA) effect size (LEfSe) indicated that *Lactobacillus* was depleted in ABX-treated mice (Fig. 3L), which is the predominant producer of AhR ligands (37), and opportunistic *Enterobacteriaceae* was enriched in ABX-treated mice (Fig. 3L). These data indicate that gut dysbiosis reduced the abundance of AhR producers but facilitated the expansion of gut pathobionts.

**FIG 4:**
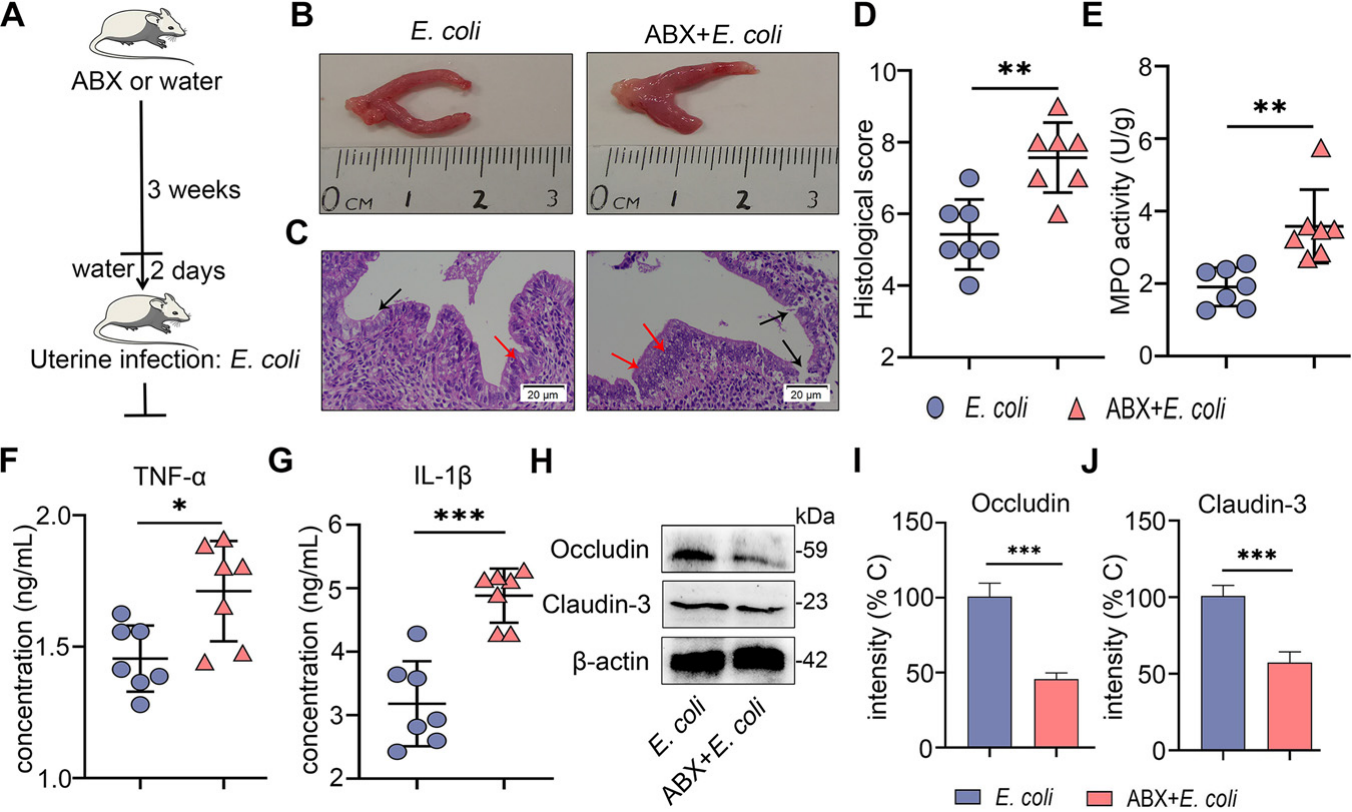
Gut dysbiosis aggravates E. coli-induced endometritis in mice. (A) A schematic representation is shown. Mice were treated with ABX (1 g/L ampicillin, metronidazole, and neomycin sulfate and 0.5 g/L vancomycin) for 3 weeks followed by E. coli administration, and uterine samples were harvested for assessment 24 h after E. coli treatment. (B-C) Representative macroscopic images (B) and H&E-stained uterine sections (C) are shown. The red arrow shows inflammatory changes (immune cell infiltration or tissue proliferation). The black arrow indicates endometrial damage (scale bar = 20 μm). (D-G) Histological score (D), MPO activity (E), and inflammatory cytokines TNF-α (F) and IL-1β (G) were determined from different treatment groups (*n* = 7). (H) Uterine occludin and claudin-3 protein expressions were assessed by Western blotting from indicated mice. Intensity analysis of occludin (I) and claudin-3 (J) was performed based on Western blotting (*n* = 7). Data are expressed as the mean ± SD, and two-tailed Student's *t* test was performed for statistical analysis (D-G and I-J). *, *P < *0.05; **P < 0.01; and ***, *P < *0.001 indicate significant differences.

We further found that ABX treatment caused more edema and congestion of the uterus compared with the control treatment (Fig. S2A). ABX treatment induced barrier injury and inflammatory cell infiltration compared with the control group, as shown by hematoxylin and eosin (H&E) staining (Fig. S2B and 2C). To confirm these results, we determined the main proinflammatory cytokines TNF-α and IL-1β and found that ABX-treated mice had expanded cytokine production (Fig. S2D). Similarly, ABX-treated mice had enhanced MPO activity compared with control mice (Fig. S2E). Moreover, lower levels of occludin and claudin-3 were observed in the ABX treatment group than the control group (Fig. S2F–H). To investigate whether gut dysbiosis aggravated E. coli-induced endometritis, an E. coli-induced endometritis model was established in the context of ABX-induced gut dysbiosis (Fig. 4A). Interestingly, E. coli-treated gut-dysbiotic mice developed more serious endometritis than E. coli-treated conventional mice, as shown by increased uterine macroscopic inflammatory changes (Fig. 4B), inflammatory infiltration and barrier disruption (Fig. 4C and D), and inflammatory markers including MPO activity (Fig. 4E), TNF-α (Fig. 4F), and IL-1β (Fig. 4G) in the E. coli-treated gut-dysbiotic mice compared with conventional mice. We also found that E. coli-treated gut-dysbiotic mice had lower uterine occludin and claudin-3 protein levels than E. coli-treated conventional mice (Fig. 4H to J). Taken together, these results demonstrate that gut microbiota disruption impairs AhR activation and facilitates E. coli-induced endometritis in mice.

### AhR ligand production by gut microbial Trp metabolism ameliorates E. coli-induced endometritis.

**FIG 5:**
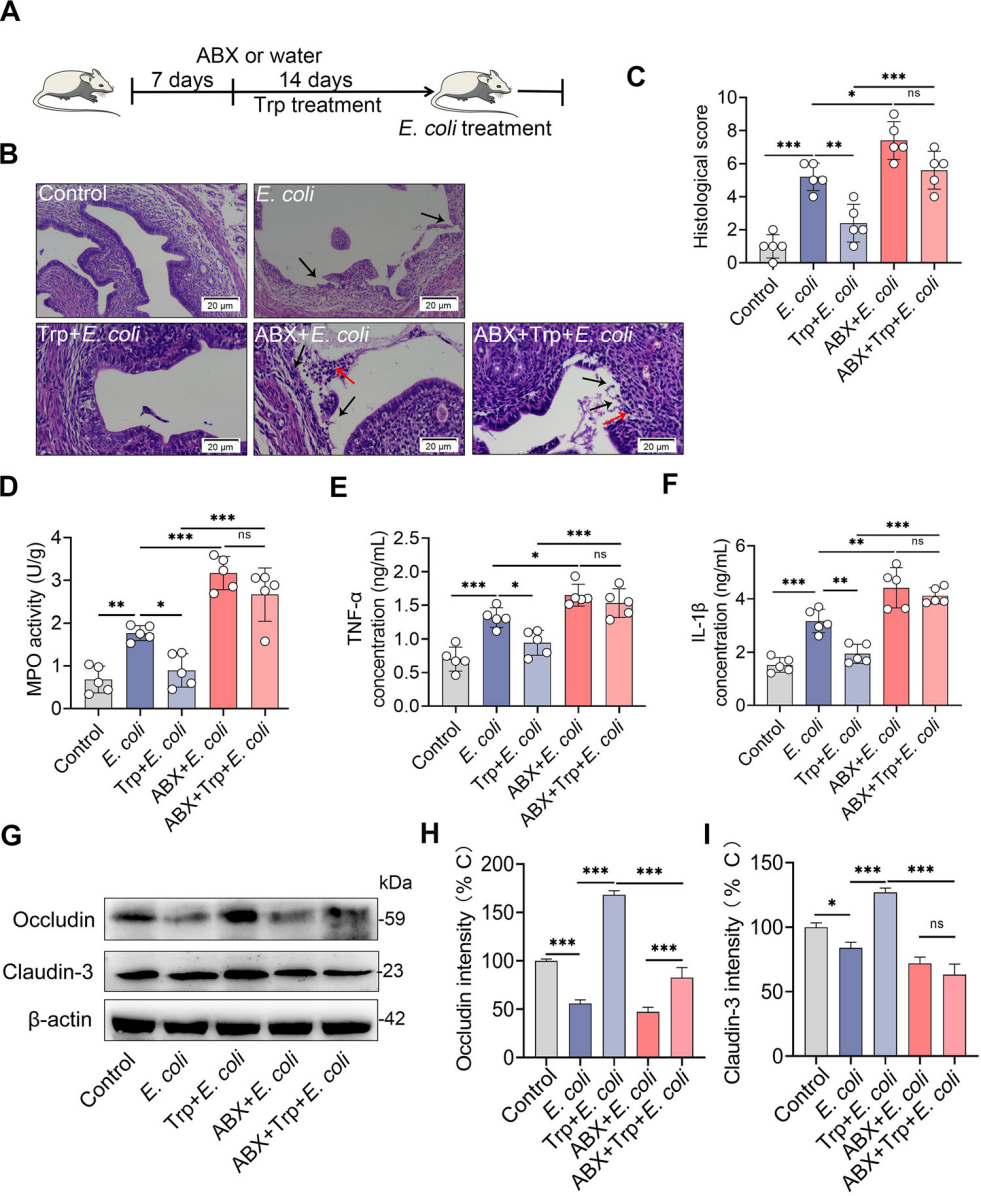
Dietary tryptophan intervention ameliorates E. coli-induced endometritis in mice in a microbiota-dependent manner. (A) Schematic representation of Trp intervention is shown. Mice were treated with ABX or water for 1 week, followed by Trp (1% added in diet) consumption for 14 days in the context with or without ABX treatment. The mice were next treated with E. coli (10^8^ CFU/30 μL on each side of the uterus) to induce endometritis after anesthetizing by urethane (100 mg/kg) intraperitoneally. (B) Representative H&E-stained uterine sections are shown. The red arrow shows inflammatory changes and the black arrow shows endometrial damage (scale bar = 20 μm). (C-F) Histological score (C), MPO activity (D), and TNF-α (E) and IL-1β (F) levels from the indicated mice were determined (*n* = 5). (G) Levels of uterine occludin and claudin-3 protein in different treatment groups were assessed (*n* = 5). Intensity analysis of occludin (H) and claudin-3 (I) was performed based on Western blotting (*n* = 5). Data are expressed as the mean ± SD, and one-way ANOVA was performed for statistical analysis (C-F and H-I). *, *P < *0.05; **, *P < *0.01; and ***, *P < *0.001 indicate significant differences. ABX, cocktail of antibiotics; Trp, tryptophan.

Microbiota-associated AhR ligands derived from microbial Trp metabolism activate AhR signaling pathways (24, 43). We investigated whether an enriched Trp diet would improve E. coli-induced endometritis. Mice were fed a diet containing high Trp (1% in diet) for 2 weeks with or without gut microbiota depletion, and then the E. coli-induced endometritis model was established (Fig. 5A). We found that Trp treatment alleviated E. coli-induced macroscopic inflammatory changes (Fig. S3). To confirm this result, histological analysis of H&E-stained sections was performed. Increased neutrophil infiltration and barrier injury were observed in the E. coli treatment group compared with the control group (Fig. 5B and C), while Trp treatment attenuated E. coli-induced uterine inflammatory changes compared with that of E. coli-treated mice (Fig. 5B and C). However, the ABX + Trp treatment group showed similar inflammatory infiltration and barrier damage compared with E. coli-treated gut-dysbiotic mice (Fig. 5B and C). Consistently, Trp-treated conventional mice had reduced inflammatory markers including MPO activity (Fig. 5D) and TNF-α (Fig. 5E) and IL-1β (Fig. 5F) levels compared with the E. coli group, but no significant differences were detected in these inflammatory markers between Trp-treated gut-dysbiotic mice and gut-dysbiotic mice upon E. coli infection (Fig. 5D–F). Moreover, Trp treatment improved the E. coli-induced decrease in the expression of the TJ proteins occludin and claudin-3 compared with the E. coli group (Fig. 5G–I). However, the protective effect of Trp on the uterine barrier was weakened after depletion of the gut microbiota (Fig. 5G–I). These results suggest that a Trp-enriched diet improves E. coli*-*induced endometritis in a gut microbiota-dependent manner.

**FIG 6:**
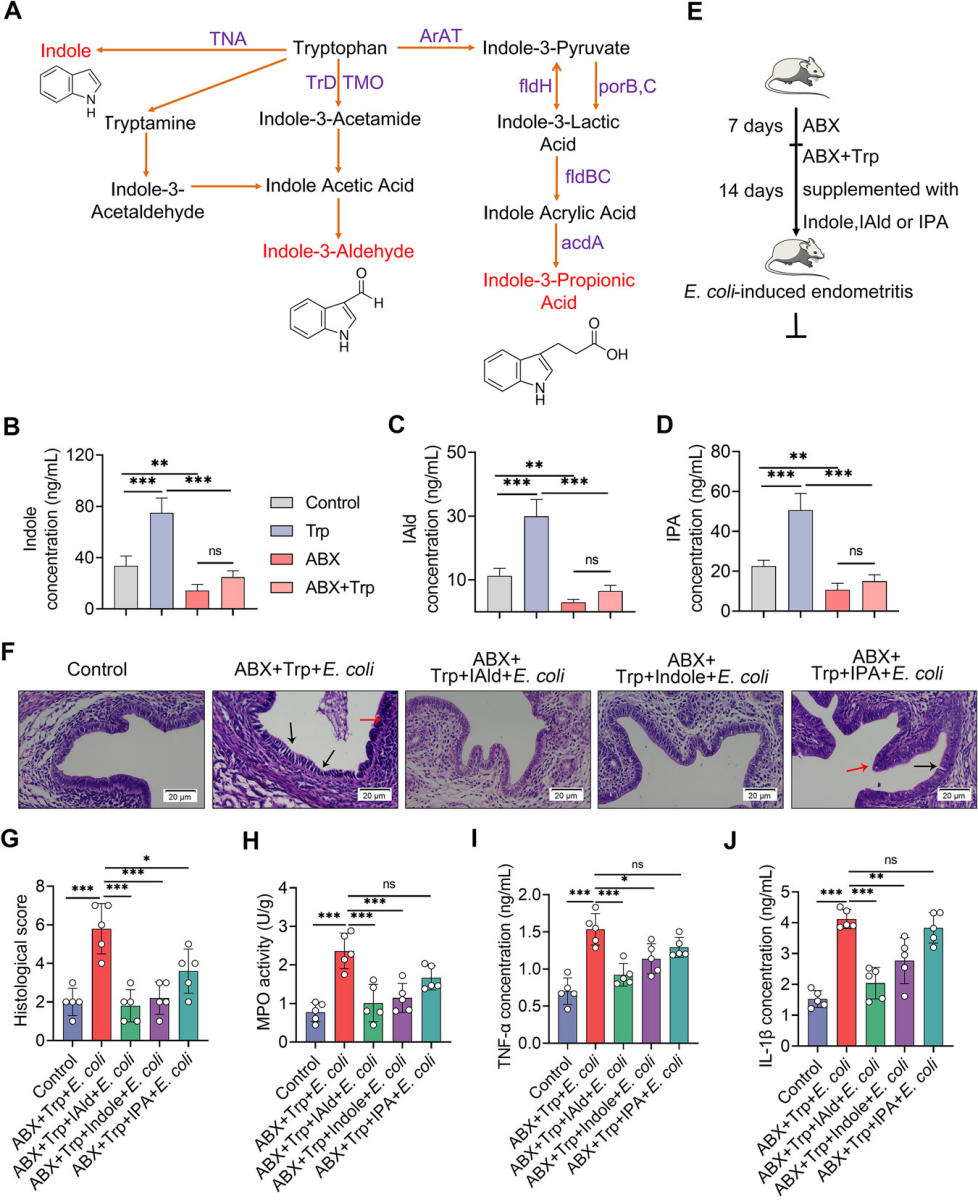
Supplementation with Trp-derived AhR ligands rescues the protective effects of Trp on E. coli-induced endometritis. (A) Microbiota-mediated Trp metabolism pathways are shown. (B-D) Intestinal indole-3-aldehyde (IAld; B), indole (C), and indole-3-propionic acid (IPA; D) levels were determined from different treatment groups (*n* = 5). (E) A schematic representation of AhR ligand supplementation is shown. Mice were pretreated with ABX for 1 week and then treated with Trp (1% in diet) and ABX for the next 2 weeks. IAld, indole, or IPA were supplemented during Trp and ABX treatment. Control mice were treated with ABX and vehicle of indole derivatives (0.2% sodium carboxymethylcellulose and 0.25% polysorbate-80 in PBS). (F) Representative H&E-stained images of uterine sections are shown. The red arrow shows inflammatory changes and the black arrow shows endometrial damage (scale bar = 20 μm). (G-J) Histological scores (G), MPO activity (H), and TNF-α (I) and IL-1β (J) levels were assessed in different treatment groups (*n* = 5). Data are expressed as the mean ± SD, and one-way ANOVA was performed for statistical analysis (B-D and G-J). *, *P < *0.05; **, *P < *0.01; and ***, *P < *0.001 indicate significant differences. ns, no significance; ABX, cocktail of antibiotics; IAld, indole-3-aldehyde; IPA, indole-3-propionic acid; Trp, tryptophan; IAld, indole-3-aldehyde; IPA, indole-3-propionic acid; acdA, acyl-CoA dehydrogenase; AraT, aromatic amino acid aminotransferase; fldBC, phenyllactate dehydratase; fldH, phenyllactate dehydrogenase; porB, C, pyruvate: ferredoxin oxidoreductase B and C; TMO, tryptophan 2-monooxygenase; TNA, tryptophanase; TrD, tryptophan decarboxylase.

Intestinal Trp metabolism follows three major pathways, including the IDO1-mediated kynurenine pathway (KP), Trp hydroxylase 1-mediated serotonin production pathway, and direct AhR ligand production by intestinal microbiota metabolism (37, 43). Indole and many indole derivatives, including indole-3-aldehyde (IAld) and indole-3-propionic acid (IPA), are among the direct metabolites of Trp into several AhR agonists by microbiota (Fig. 6A), and these derivatives account for the predominant AhR activation *in vivo* (24, 37, 43, 51). Given that alternation of intestinal microbiota regulated AhR activation (Fig. 3A–C), deteriorated pathogen-induced endometritis (Fig. 4A–J), and reversed Trp protective effects in gut-dysbiotic mice (Fig. 5A-J), we then tested whether AhR ligand production by gut microbial Trp metabolism accounted for the protective effects of Trp against E. coli*-*induced endometritis. We first showed that Trp supplementation increased intestinal levels of indole (Fig. 6B), IAld (Fig. 6C), and IPA (Fig. 6D) compared with the control group, while ABX-induced gut-dysbiotic mice had lower intestinal levels of indole derivatives (Fig. 6B–D). Consistently, our results confirmed that the production of the AhR ligands indole, IAld, and IPA required the gut microbiota, which was evidenced by the reduced intestinal level of indole derivatives in Trp-treated gut-dysbiotic mice compared with Trp-treated conventional mice (Fig. 6B-D). Next, mice were treated with IAld, indole, or IPA in the context of ABX and Trp treatment (Fig. 6E). We found that IAld, indole, and IPA treatment improved uterine pathological injury by reducing inflammatory infiltration and improving barrier integrity compared with the ABX and Trp treatment groups (Fig. 6F and G; Fig. S4A). Indole and IAld, but not IPA, treatment groups, had lower levels of MPO activity (Fig. 6H), TNF-α (Fig. 6I), and IL-1β (Fig. 6J) than ABX-Trp-treated mice, and higher levels of uterine TJ protein occludin and claudin-3 (Fig. S4B–D), which suggests that supplementation of AhR ligands rescued the protective effects of Trp on E. coli-induced endometritis. Similar to previous reports that indole and IAld had a stronger capacity for AhR activation and more effective anti-inflammatory effects on autoimmune encephalomyelitis (EAE) (52), these results suggest that AhR activation and subsequent inflammation regulation depend on ligand affinity (27, 43, 52). We also found that IAld, indole, and IPA treatment alleviated E. coli-induced endometritis, which was shown by improved uterine inflammatory injury (Fig. S5A–B) and reduced inflammatory markers including MPO activity (Fig. S5C), TNF-α (Fig. S5D), and IL-1β (Fig. S5E) compared with the E. coli group. Collectively, these results indicate that AhR ligand production by microbial Trp metabolism improves E. coli*-*induced endometritis through inflammation limitation and barrier repair.

### L. reuteri consumption alleviates E. coli-induced endometritis in mice in an AhR-dependent manner.

**FIG 7:**
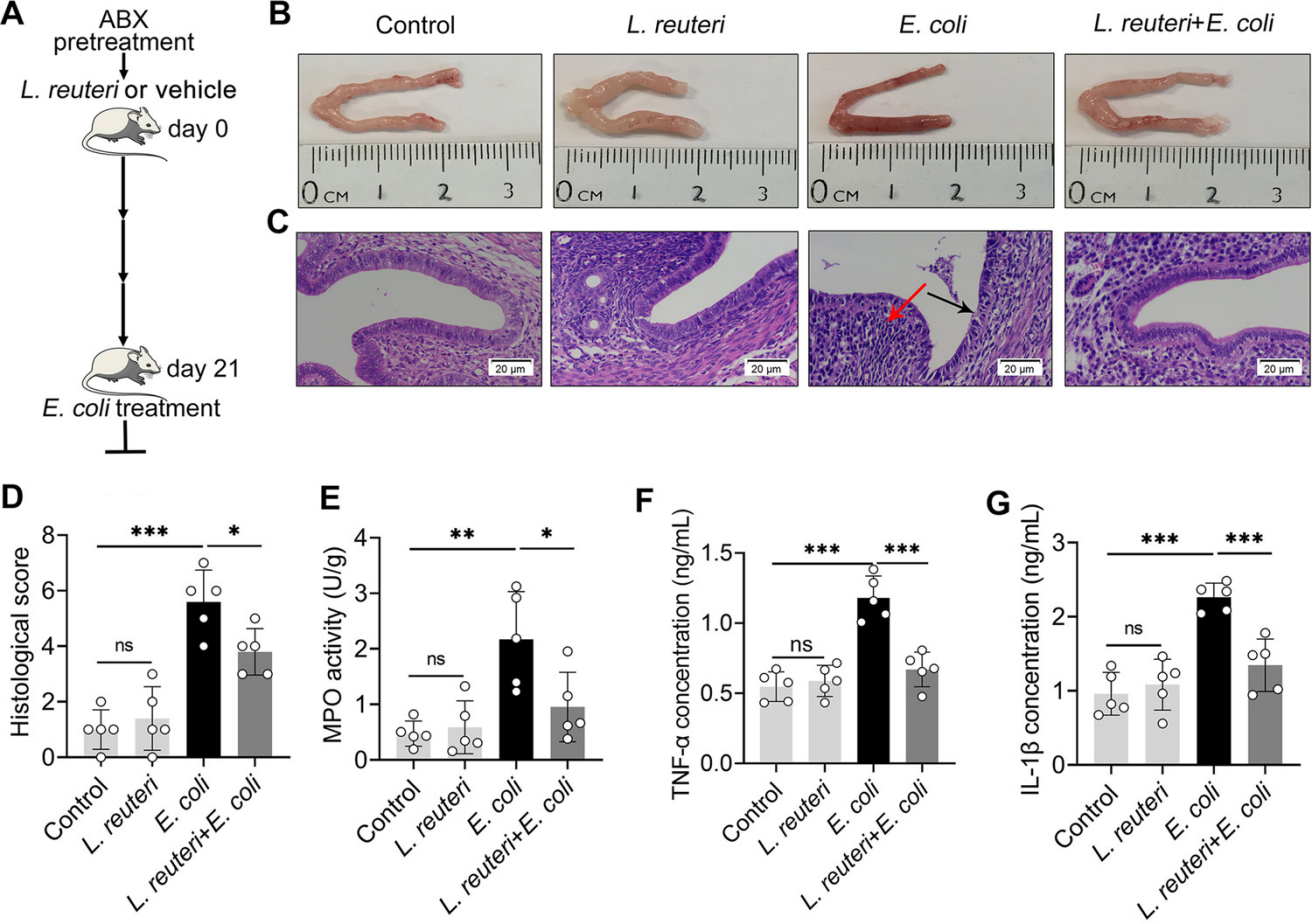
L. reuteri ameliorates E. coli-induced endometritis in mice. (A) A schematic diagram of L. reuteri treatment is shown. Mice were treated with L. reuteri (10^9^ CFU/300 μL) once every 2 days for 3 weeks after eradicating the preexisting gut commensal microbiota by ABX. On day 21, mice were treated with E. coli (10^8^ CFU/30 μL) by intrauterine injection. After 24 h, uterine tissues were collected for determinations. (B–C) Representative macroscopic (B) and H&E-stained (C) images of uteri from different treatment groups are shown. (D) Histological scores were evaluated based on the barrier damage and inflammatory cell infiltration (*n* = 5). (E-G) Uterine MPO activity and TNF-α (F) and IL-1β (G) levels were assessed in differently treated mice (*n* = 5). Data are expressed as the mean ± SD, and one-way ANOVA was performed for statistical analysis (D-G). *, *P < *0.05; **, *P < *0.01; and ***, *P < *0.001 indicate significant differences. ns, no significance.

Given that *Lactobacillus* was enriched in control mice but depleted in gut-dysbiotic mice (Fig. 3J–L), and previous findings that L. reuteri activated AhR through metabolizing Trp and affected disease outcomes (28, 37), we investigated the impact of L. reuteri consumption on *E.coli*-induced endometritis. The mice were orally gavaged with L. reuteri once every 2 days for 21 days (6, 28), followed by E. coli treatment (Fig. 7A). We first confirmed that L. reuteri successfully colonized the gut (Fig. S6A). As expected, L. reuteri treatment reduced *E.coli*-induced macroscopic inflammatory changes (Fig. 7B), endometrial epithelial injury, and inflammatory cell infiltration (Fig. 7C and D). Consistently, L. reuteri-treated mice had lower MPO activity (Fig. 7E), TNF-α (Fig. 7F), and IL-1β (Fig. 7G) levels than E. coli-treated mice. These results suggest that L. reuteri alleviates E. coli-induced endometritis in mice.

We also investigated the effects of supplementation with L. reuteri on the uterine barrier. Mice supplemented with L. reuteri had higher uterine occludin (Fig. S6B), claudin-3 (Fig. S6C), and TJP1 (Fig. S6D) gene expressions than control mice. L. reuteri pretreated mice rescued the decreases in uterine occludin (Fig. S6B), claudin-3 (Fig. S6C), and TJP1 (Fig. S6D) caused by E. coli. Consistently, L. reuteri treatment increased occludin and claudin-3 protein levels compared with control mice (Fig. S6E–G). E. coli treatment reduced uterine occludin and claudin-3 expression, while L. reuteri consumption reversed these reductions (Fig. S6E–G). We next found that L. reuteri treatment reduced the levels of p-p65 and p-IκB in the uterus compared with E. coli treatment (Fig. S7A-C). These results indicate that L. reuteri improves uterine barrier integrity and limits E. coli-induced activation of the NF-κB pathway.

**FIG 8:**
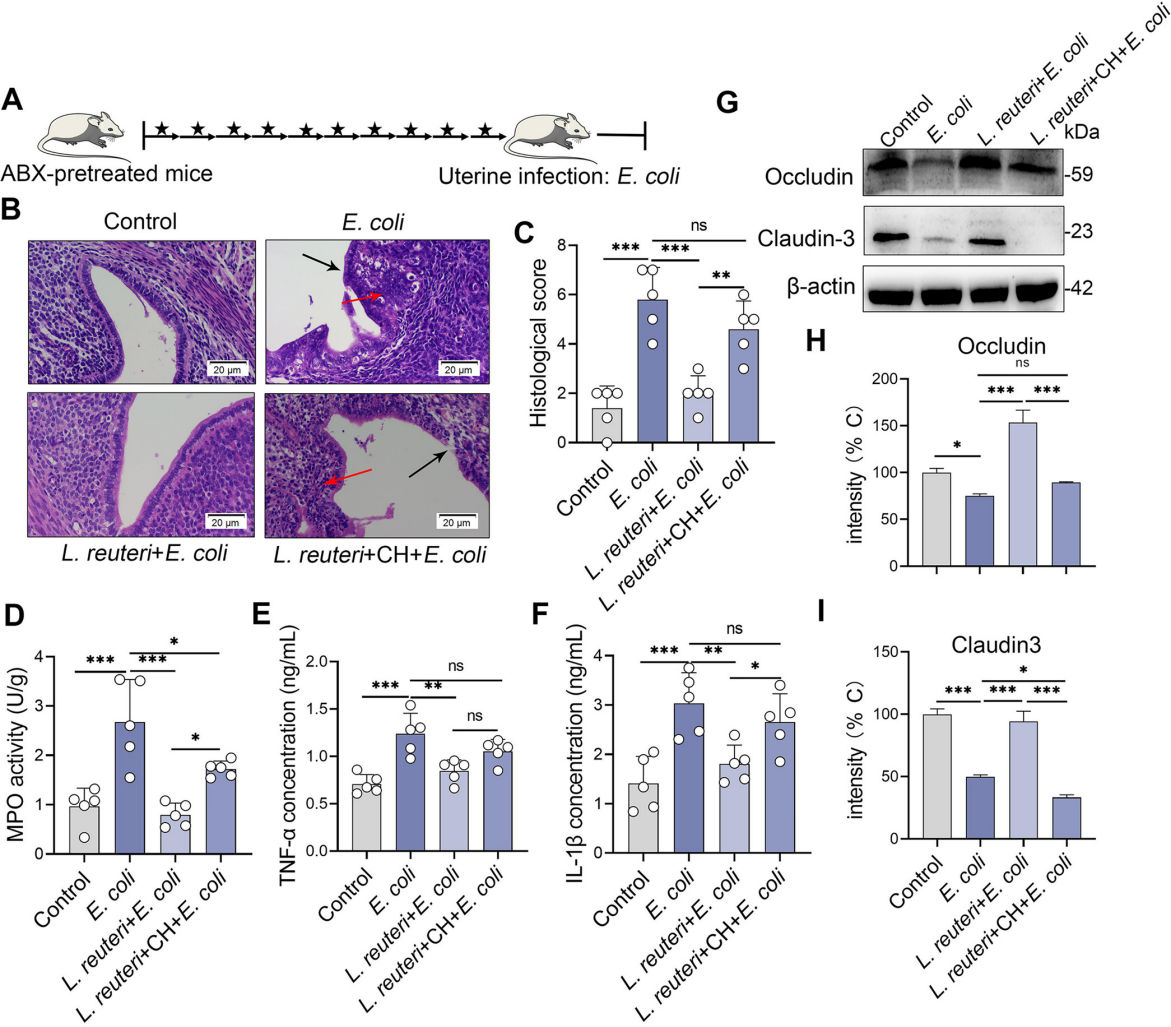
The protective effects of L. reuteri on E. coli-induced endometritis depend on the activation of AhR in mice. (A) Schematic representation of L. reuteri and CH223191 treatment is shown. Mice were treated with CH223191 (500 μg/kg BW) intraperitoneally after each oral gavage of L. reuteri (10^9^ CFU/300 μL). The arrow indicates L. reuteri supplementation and the star indicates CH223191 treatment. On day 21, mice were treated with E. coli (10^8^ CFU/30 μL) to induce endometritis. (B) Representative H&E-stained uterine images are shown. (C-F) Histological scores (C), MPO activity (D), and TNF-α (E) and IL-1β (F) levels were determined from different treatment groups (*n* = 5). (G) Levels of uterine occludin and claudin-3 from the indicated mice were assessed by Western blotting. Intensity analysis of occludin (H) and claudin-3 (I) was performed based on Western blotting (*n* = 5). Data are expressed as the mean ± SD, and one-way ANOVA was performed for statistical analysis (C-F and H-I). *, *P < *0.05; **, *P < *0.01; and ***, *P < *0.001 indicate significant differences. ns, no significance. CH, CH223191.

L. reuteri has been described as activating AhR by metabolizing dietary Trp into AhR agonists (28, 37); we then found that L. reuteri increased AhR expression (Fig. S8A). To confirm this result, we determined the levels of the AhR target genes *Cyp1a1* and *Cyp1b1*. We found that L. reuteri, but bot E. coli, increased *Cyp1a1* and *Cyp1b1* levels in mouse uterine tissues (Fig. S8B and 8C), which suggests that L. reuteri activates the AhR pathway by producing AhR agonists. Consistently, L. reuteri increased AhR (Fig. S8D and E) and Cyp1a1 protein levels with or without E. coli treatment (Fig. S8D and E). To examine whether the protective effects of L. reuteri on E. coli-induced endometritis depended on the activation of AhR, we blocked AhR by treating mice with CH223191 after each oral gavage of L. reuteri for 3 weeks (Fig. 8A). CH223191 treatment reversed the protective effects of L. reuteri on E. coli-induced macroscopic and microscopic inflammatory changes (Fig. 8B and C; Fig. S9). Moreover, the CH223191 treatment group had increased inflammatory markers including MPO activity (Fig. 8D), TNF-α (Fig. 8E), and IL-1β (Fig. 8F) compared with the L. reuteri treatment group. Consistently, AhR inhibition weakened the protective effects of L. reuteri on E. coli-induced uterine barrier disruption by reducing the expression of the TJ proteins occludin and claudin-3 (Fig. 8G–I). These results suggest that the protective effects of L. reuteri on E. coli-induced endometritis rely on AhR activation.

## DISCUSSION

The gut microbiota participates in numerous physiological functions and disease processes by affecting multiple aspects of the host through activating host receptors by microbiota-mediated metabolites (2, 3). AhR activation by microbial Trp metabolism is involved in the pathogenesis of metabolic syndrome (6), inflammatory bowel disease (28), celiac disease (42), alcohol-induced liver injury (53), candidiasis (37), and EAE (52). Impaired AhR ligand production by alteration of the gut microbiota is also implicated in the development of diseases (6, 42). Here, we tested whether gut microbiota-regulated AhR activation by Trp metabolites suppressed infection-induced inflammation using a mouse endometritis model. We found that AhR activation improved E. coli-induced endometritis by limiting inflammation expansion and restoring epithelial barrier functions. Abrogation of the gut microbiota by ABX weakened AhR activation in the uterus, induced uterine inflammation, and aggravated E. coli-induced endometritis. Moreover, supplementation with a diet enriched in high Trp ameliorated E. coli-induced endometritis by increasing ligand levels of AhR. Consumption of L. reuteri with effective AhR ligand-producing capacity also alleviated E. coli-induced endometritis in mice.

AhR is a ligand-activated transcription factor that integrates environmental, dietary, microbial, and metabolic cues to modulate intricate transcriptional programs (26, 27). Due to the wide expression of AhR in epithelial cells and most immune cells, including T cells, dendritic cells, and macrophages, (26, 27), AhR participates in many aspects of health and disease. Moreover, AhR provides a model signaling pathway to study the potential molecular mechanisms of microbial-derived metabolite regulation of the immune response in health and disease and thus to explore new strategies for disease intervention. To study the role of AhR activation in E. coli-induced endometritis immunopathology, we pharmacologically regulated the AhR signaling pathway using Ficz or CH223191. We showed that Ficz treatment alleviated E. coli-induced inflammation and barrier injury, but pretreatment with an AhR inhibitor reversed these effects. Previous studies indicated that AhR activation by the classical AhR agonist of Ficz inhibited the development of experimental colitis (28), metabolic disorders (6), EAE (52), and chronic inflammation (42) via IL-22 production or direct transcriptional regulation, which are related to the restoration of barrier function (54). Additionally, AhR activation by Ficz restored hypoxia-induced barrier dysfunction in intestinal epithelial cells *in vitro*, which was associated with increased expression of TJ proteins (54). The AhR-mediated immune tolerant state caused by LPS stimulation protected the host against Gram-negative and Gram-positive pathogen infections, which suggests the essential role of AhR activation in inflammatory diseases (44). Previous studies also indicated that mice with AhR deficiency had higher susceptibility to Citrobacter rodentium (C. rodentium) (47, 55, 56), which is a model that mimics human E. coli infections. Upon LPS stimulation, an increased inflammatory state was detected in AhR-deficient mice, which is consistent with increased p65 levels in the nucleus, indicating that AhR activation has direct effects on NF-κB signaling transcription and limiting inflammation expansion (44, 52, 55). E. coli infection is one of the main causes of endometritis (34, 35), and E. coli or LPS can activate AhR signaling that regulates the host inflammatory response and barrier integrity (30, 32).

AhR pathway activation participates in many aspects of host homeostasis, especially intestinal microbiota-mediated physiological and pathogenic progresses (20, 24, 43). AhR can be activated by numerous endogenous ligands, most of which are derived from Trp metabolism by the host and microbiota (26, 43). Host-mediated AhR ligands, including kynurenine, kynyrenic acid, xanthurenic acid, and cinnabarinic acid, are produced by IDO1-regulated KP in immune and epithelial cells (43). Microbiota-mediated AhR ligands, including indol-3-acetic acid, indole, IAld, and IPA, are indole and its derivatives (26, 43). Some AhR ligands, such as indoxyl-3-sulfate, are produced by the integration of host and microbial metabolism (26). Germ-free or gut-dysbiotic mice have deficient AhR ligands and more severe colitis score (28). ABX-treated mice showed increased EAE symptoms and decreased AhR ligand production and L. reuteri levels (52). Using ABX-treated mice, we showed that gut-dysbiotic mice developed uterine inflammation and endometrial barrier function damage, which are consistent with AhR activation impairment in the uterus and may be due to the abrogation of the intestinal microbiota (18). Similar to our previous study (18), aggravated inflammatory status was also observed in ABX-pretreated mice followed by E. coli stimulation, which suggests that impairment of AhR activation in the uterus by the gut microbiota alteration facilitates the progression of E. coli-induced endometritis.

Dietary Trp metabolism by the gut microbiota accounts for the main ligand production of AhR (20, 43). Supplementation with Trp was sufficient to reduce the disease scores of endometritis but reversed in the context of gut microbiota abrogation. Impairment of microbial Trp metabolism in gut-dysbiotic mice reduced AhR ligand levels and promoted the KP pathway of Trp metabolism (24, 43), which positively correlated with IBS (irritable bowel syndrome) severity (57). Treatment with IAld, indole, and IPA rescued the protective role of Trp to different degrees, which may depend on different capacities of AhR activation (43, 52). Consistently, an indole-3-carbinol supplemented diet reduced C. rodentium burden and promoted mouse survival (58), which led to the enhanced AhR ligand levels supplemented by dietary Trp being sufficient to activate the AhR pathway and regulate disease development. L. reuteri metabolizes dietary Trp into several AhR agonists (28, 37). We found that L. reuteri supplemented mice had higher AhR activation in the uterus. A previous study demonstrated that L. reuteri improved the outcomes of colitis, but AhR inhibition reversed this improvement (28). Other studies also indicated that L. reuteri ameliorated metabolic syndrome, celiac disease, and alcohol-induced liver injury by activating AhR (6, 42, 53). Inhibition of AhR activation pharmacologically weakened the protective effects of L. reuteri on inflammation limitation and barrier repair. However, we cannot exclude that L. reuteri improved the outcome of endometritis in other manners. Further studies should investigate other potential mechanisms by which L. reuteri activates AhR to improve endometritis or other diseases and the possibility of a combined treatment of Trp and L. reuteri to improve endometritis.

Altogether, our study identified that gut-dysbiotic mice had increased severity of endometritis, which was associated with impaired AhR activation. E. coli-induced endometritis was rescued by pharmacological activation of AhR, dietary Trp intervention, and supplementation with AhR ligands and producer. Because there are few treatments that sufficiently improve uterine infection other than antibiotics, which show increased resistance and risk of infection recurrence, modulation of the AhR pathway using a combination of pharmacological AhR ligands, diet, prebiotics, and AhR ligand-producing bacteria may be a new preventative or therapeutic strategy for endometritis and other infectious or metabolic diseases.

## MATERIALS AND METHODS

### Animals.

All specific pathogen free (SPF) grade BALB/c mice were bought from Liaoning Changsheng Biotechnology Co., Ltd. (Benxi, China). Mice were supplemented with enough water and breeding fodder in the condition, with 12 h of light daily. All animal experiments were approved by the Institutional Animal Care and Use Committee (IACUC) of Jilin University. The full proposal was reviewed by the IACUC ethics committee, which approved the animal care and use permit license. All experiments complied with the manual of the care and use of laboratory animals published by the U.S. National Institutes of Health.

### Materials.

The main regents used in this study, Trp, indole, IPA, IAld, Ficz, CH223191, metronidazole, ampicillin, vancomycin, and neomycin sulfate, were purchased from Sigma-Aldrich (St. Louis, MO, USA). The specific primary antibodies of AhR and Cyp1a1 were bought from Affinity Biosciences (OH, USA). Phosphorylation (p-) of p65 and IκB, p65, IκB, and β-actin were obtained from Cell Signaling Technology (CST; Boston, USA). The occludin and claudin-3 were bought from Bioss (Beijing, China). MPO activity assay kit was purchased from Nanjing Jiancheng Bioengineering Institute (Nanjing, China). TNF-α and IL-1β enzyme linked immunosorbent assay (ELISA) kits were bought from Biolegend, Inc. (San Diego, CA, USA). Indole and IAld ELISA kits were bought from Hnybio (Shanghai, China), and the IPA ELISA kit was obtained from Shyqbio (Shanghai, China). L. reuteri CNCM I-5022 was obtained from the Collection Nationale de Cultures de Microorganisms (CNCM) of the Institut Pasteur. *E.coli* CVCC1418 was obtained from the China Veterinary Culture Collection Center (CVCC).

### Antibiotics cocktail treatment.

Mice were treated with an antibiotic cocktail consisting of metronidazole (1 g/L), ampicillin (1 g/L) and neomycin sulfate (1 g/L), and vancomycin (0.5 g/L) in drinking water for 3 weeks (9). ABX was removed from the drinking water for 2 days before establishing a model of E. coli-induced endometritis.

### AhR agonist and antagonist treatments.

In experiments treated with AhR agonist or antagonist, 2-methyl-2H-pyrazole-3-carboxylic acid (CH223191; 500 μg/kg body weight [BW]) were pretreated 1 h intraperitoneally before Ficz application (49, 59). Ficz was dissolved in dimethyl sulfoxide (DMSO) as previously mentioned (59). Finally, Ficz (50 μg/kg BW) was administered via intraperitoneal injection 1 h before E. coli stimulation (59). Control mice were treated with equal DMSO.

### Trp and Trp metabolites supplement experiments.

To assess the effects of Trp on endometritis, mice were treated with a control diet (AIN93G) and supplemented with Trp (1% in diet) for 2 weeks (45, 60, 61), with or without ABX pretreated for a week and then treated throughout experiment (52). For Trp derivatives rescue experiments, indole, IPA, and IAld (20 mg/kg BW) were orally gavaged to mice for 14 consecutive days in the context of Trp and ABX treatment (52). The control group was treated with ABX and a vehicle for indole derivatives (0.2% sodium carboxymethylcellulose and 0.25% polysorbate-80 in phosphate-buffered saline [PBS]) (45). In addition, to investigate the protective effects of AhR ligands on endometritis, subjects were also treated for 14 consecutive days with indole, IPA, and IAld (20 mg/kg BW), and then treated with E. coli.

### L. reuteri treatments.

Before L. reuteri was given by oral gavage, mice were pretreated to eradicate the commensal gut microbiota using ABX in the drinking water as previously described (62). The last day before gavage, antibiotics were removed from the drinking water. In experiments evaluating the effect of L. reuteri on E. coli-induced endometritis, mice were orally gavaged L. reuteri (10^9^ CFU/300 μL) once every 2 days for 21 days (6, 28, 37, 42). Mice in the control group were orally gavaged an equal vehicle (MRS broth supplemented with 0.05% l-cysteine and 15% glycerol) (6, 42). For the experiment to detect the AhR dependence of L. reuteri, mice were simultaneously treated with CH223191 (500 μg/kg BW) intraperitoneally after each oral L. reuteri (28).

### Mouse endometritis model.

The experimental endometritis model was induced using an E. coli CVCC1418 strain as previously described (15, 16, 18). Briefly, E. coli CVCC1418 was cultured in lysogeny broth (LB; Haibo, Qingdao, China) at 37°C 180 rev/min. Mice were anesthetized by using urethane (100 mg/kg) intraperitoneally and an intrauterine injection of E. coli (10^8^ CFU/30 μL) by using a 100-μL syringe with a 30-gauge blunt needle. The mice were sacrificed 24 h after model induction, and uterine tissues were collected and stored at −80°C until detection.

### Histological evaluation of the uterus.

All mice uterus samples for histology assessment were treated with 4% paraformaldehyde, then embedded in paraffin and prepared for 5-μm paraffin sections (three sections per sample). Paraffin sections were stained with H&E and then detected using an optical microscope (BX51 Olympus, Tokyo, Japan). Histological score was performed according to a combination of inflammatory cell infiltration (graded 0–3, from none to severe), hyperplasia (graded 0–3, from none to severe), and epithelial barrier disruption (graded 0–3, from normal to severe damage) (15, 18).

### MPO activity determination.

As the specific marker of neutrophils, MPO was generally detected to reflect the degree of neutrophil infiltration. In order to appraise the MPO levels, 10% tissue homogenates were prepared and MPO activity was calculated according to the manufacturer's instruction (A044-1-1, Nanjing Jiancheng, China).

### Cytokines assays.

To determine the inflammatory cytokine expressions, 10% tissue homogenates from uterine tissues were prepared using PBS, and ELISA kits for TNF-α (cat. #430901, Biolegend, USA) and IL-1β (cat. #432601, Biolegend, USA) were used according to manufacturer’s instruction. The concentration of cytokines was calculated according to the standard curve.

### Quantification of fecal AhR ligands.

The fecal samples were harvested from Trp treatment groups. All fecal pellets from each group were weighted and diluted with PBS at the finial concentration of 100 mg/mL, and fecal AhR ligands, including indole, IAld, and IPA, were measured as previously described (63). In brief, the stool was fully dissolved and centrifuged at 800 rpm for 3 min. The supernatant was collected to determine the level of AhR ligands, including indole (Hnybio, Shanghai, China), IAld (Hnybio, Shanghai, China), and IPA (Shyqbio, Shanghai, China), using an ELISA kit according to the manufacturer's protocol.

### RNA extraction and qPCR.

Uterine tissues were treated with TRIzol (1 mL/100 mg) and RNA, was extracted as previously described (64). In brief, TRIzol-treated solution was treated with 200 μL chloroform and 500 μL isopropanol and washed with 75% DEPC water-diluted alcohol. After dissolving with DEPC water, RNA was reversely transcribed into cDNA using TransScript One-Step gDNA Removal and cNDA Synthesis SuperMix (Transgen Biotech, Beijing, China). SYBR green master (Roche, Germany) with mouse specific primer in a StepOnePlus apparatus (Applied Biosystems, Foster City, CA, USA) was performed. The oligonucleotides used are shown in Table S1. 2^-△△Ct^ quantification methods were used and GAPDH served as an endogenous control.

### Western blotting.

Total protein samples were collected by a tissue protein extract (Thermo Fisher Scientific, USA), and protein concentrations were measured using a BCA protein assay kit (Thermo Fisher Scientific, USA). Targeting proteins were separated using 10% SDS-PAGE based on molecular size, and then proteins were bonded to 0.45-μm polyvinylidene fluoride (PVDF) membranes. After blocked in 5% skim milk for 3 h at room temperature, PVDF membranes were incubated with specific primary antibody (1:1000 for AhR, Cyp1a1, p-p65, p-IκB, p65, IκB, and β-actin) at 4°C overnight. Further, PVDF membranes were incubated with goat anti-rabbit IgG (1:20,000) for 2 h at room temperature after washing three times with TBST (Tris-buffered saline with Tween 20). Finally, proteins were tested using ECL plus the Western blotting detection system.

### Immunohistochemistry.

Uterus tissues were processed as previously described (64). In brief, sections were treated by xylene for 30 min 100%, 95%, and 80% alcohol for 5 min. Each treatment was repeated twice. After antigen retrieval using sodium citrate and washing by phosphate (PBS), uterus sections were incubated with endogenous peroxidase blockers (UltraSensitive SAP [Mouse/Rabbit] IHC Kit, MXB, China) for 40 min and blocked with 5% goat serum for 30 min at room temperature, then incubated with antibody against AhR (rabbit anti-AhR, Affinit) or Cyp1a1 (rabbit anti-AhR, Affinit) at 4°C overnight. Sections were incubated with secondary antibody (goat anti-rabbit IgG) at room temperature for 40 min after washing by PBS. Sections were developed under a microscope using a color developing agent (UltraSensitive SAP [Mouse/Rabbit] IHC Kit, MXB, China) and terminated by water after horseradish peroxidase (HRP) treatment. Hematoxylin was used for nucleus staining followed by 1% muriatic acid alcohol and ammonium hydroxide treatment. The positive staining was assessed using an optical microscope.

### Microbial DNA extraction, PCR amplification, and Miseq sequencing in fecal contents.

Genomic DNA amplification and sequencing were performed as previously mentioned (9). Briefly, fecal microbial DNA was extracted using the cetyltrimethylammonium bromide (CTAB) method. The 16S rRNA V4 region of the eukaryotic rRNA gene was amplified by PCR using primers 515F–806R. Sequencing libraries were generated using the TruSeq DNA Sample Prep Kit (Illumina, USA) and sequenced using an IIIuminaHiSeq2500 platform, and 250 bp paired-end reads were generated. Paired-end reads were merged using FLASH (v1.2.7; http://ccb.jhu.edu/software/FLASH/) (65). Quality filtering on the raw tags was performed under specific filtering conditions to obtain the high-quality clean tags according to the QIIME quality control process (v1.9.1; http://qiime.org/scripts/split_libraries_fastq.html) (66). The obtained 16S rRNA sequences are shown in Table S2. Sequence analysis was performed by Uparse software (Uparse v7.0.1001; http://drive5.com/uparse/). Alpha diversity, including the observed species, Shannon, Chao1, and ace index, was calculated with QIIME. PCoA analysis was displayed by the WGCNA package, stat packages, and ggplot2 package in R software (version 2.15.3). LEfSe analysis (log_10_ LDA score > 4) was used to identify significantly different bacterial taxa enriched in indicated groups (67).

### Bacterial culture and preparation.

L. reuteri CNCM I-5022 was grown in MRS (Haibo, Qingdao, China) broth with 0.05% l-cysteine in anaerobic conditions for 48 h (6). E. coli was grown in LB (Haibo, Qingdao, China) for 12 h to reach the mid logarithmic period.

### Quantification of L. reuteri DNA in mouse feces.

Bacterial DNA in 200 mg of fecal samples from control or L. reuteri treatment mice was purified using TIANamp Stool DNA Kit (TIANGEN) and subjected to SYBR green qPCR using primers specific to L. reuteri as previously described (52). L. reuteri primers used are in Table S1.

### Statistical analysis.

GraphPad Prism 8 (San Diego, CA, USA) was used for statistical analyses. Data are expressed as the mean ± SD. For comparison between two groups, two-tailed Student's *t* test or Mann-Whitney *U* test was performed. One-way analysis of variance (ANOVA) was performed for comparison of multiple groups. *P < *0.05 indicates statistical significance. Statistical methods used are indicated in figure legends. The displayed data are from one representative experiment out of three independent experiments.

### Data availability.

The 16S rRNA gene sequencing data in the present study are available in the NCBI Sequence Read Archive (SRA) repository under accession number PRJNA835218.
