# Supplementary material for: Dietary Tryptophan-Mediated Aryl Hydrocarbon Receptor Activation by the Gut Microbiota Alleviates Escherichia coli-Induced Endometritis in Mice
Source: Microbiol Spectr. 2022 Jun 21;10(4):e00811-22. doi: 10.1128/spectrum.00811-22 (PMC9430277; doi:10.1128/spectrum.00811-22)
Supplement: Supplemental file 1 — Supplemental material. Download spectrum.00811-22-s0001.pdf, PDF file, 1.2 MB [file spectrum.00811-22-s0001.pdf]

Supplementary files for

Dietary tryptophan-mediated aryl hydrocarbon receptor activation by the gut microbiota alleviates *Escherichia coli*-induced endometritis in mice

Caijun Zhao[1], Lijuan Bao[1], Min Qiu[1], Lianjun Feng[1], Luotong Chen[1], Zhuoyu Liu[1], Shiyu Duan[1], Yihong Zhao[1], Keyi Wu[1], Naisheng Zhang[1], Xiaoyu Hu[1]*, Yunhe Fu[1]*

1.Department of Clinical Veterinary Medicine, College of Veterinary Medicine, Jilin University, Changchun, Jilin Province 130062, China.

*** Corresponding author:**

Yunhe Fu, E-mail: fuyunhesky@163.com.

Xiaoyu Hu, E-mail: hxiaoyu@yeah.net.

This file includes:

Supplementary figures S1-S9

Supplementary table S1

Supplementary figures S1-S9

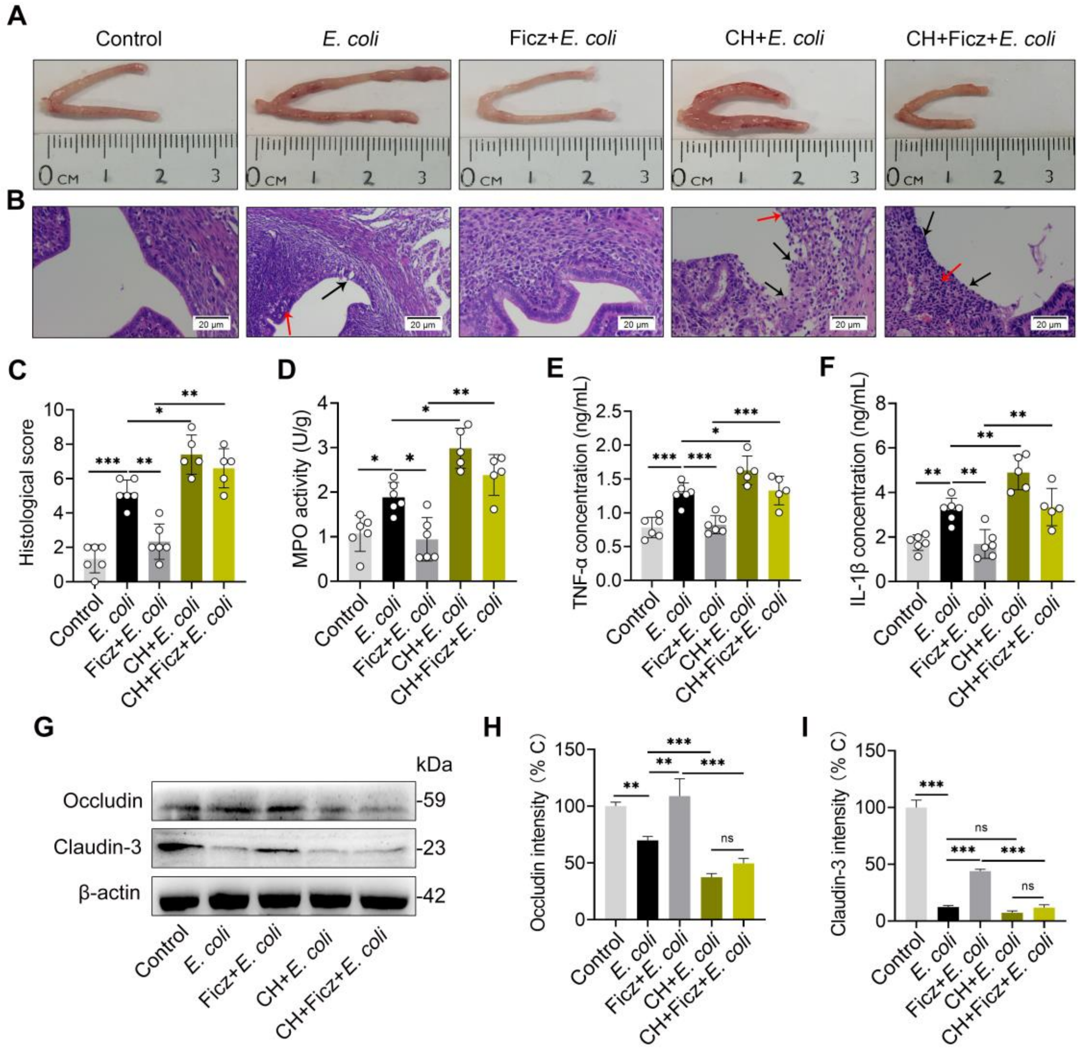

Fig. S1 CH223191 treatment aggravates *E. coli*-induced endometritis. Mice were pretreated with CH223191 (500 μg/kg BW) intraperitoneally 1 h before Ficz (50 μg/kg BW) administration, followed by *E. coli* stimulation (10^8 CFU/30 μL on each side of the uterus). **A**. Representative macroscopic images from indicated groups are shown. **B**. Representative H&E-stained images of uterine sections are shown. The black arrow indicates endometrial injury and the red arrow shows inflammatory cell infiltration (scale bar=20 μm) (n=5-6). **C-F**. Histological score (**C**), MPO activity (**D**), pro-inflammatory cytokines of TNF-α (**E**) and IL-1β (**F**) were assessed (n=5-6). **G**. Levels of uterine occludin and claudin-3 were assessed by western blotting (n=5). Intensity analysis of occludin (**H**) and claudin-3 (**I**) was performed based on western blotting (n=5). Data are expressed as the mean ± SD and one-way ANOVA (**C-F** and **H-I**) was performed. *$p < 0.05$, **$p < 0.01$ and ***$p < 0.001$ indicate significant differences. ns, no significance. CH, CH223191.

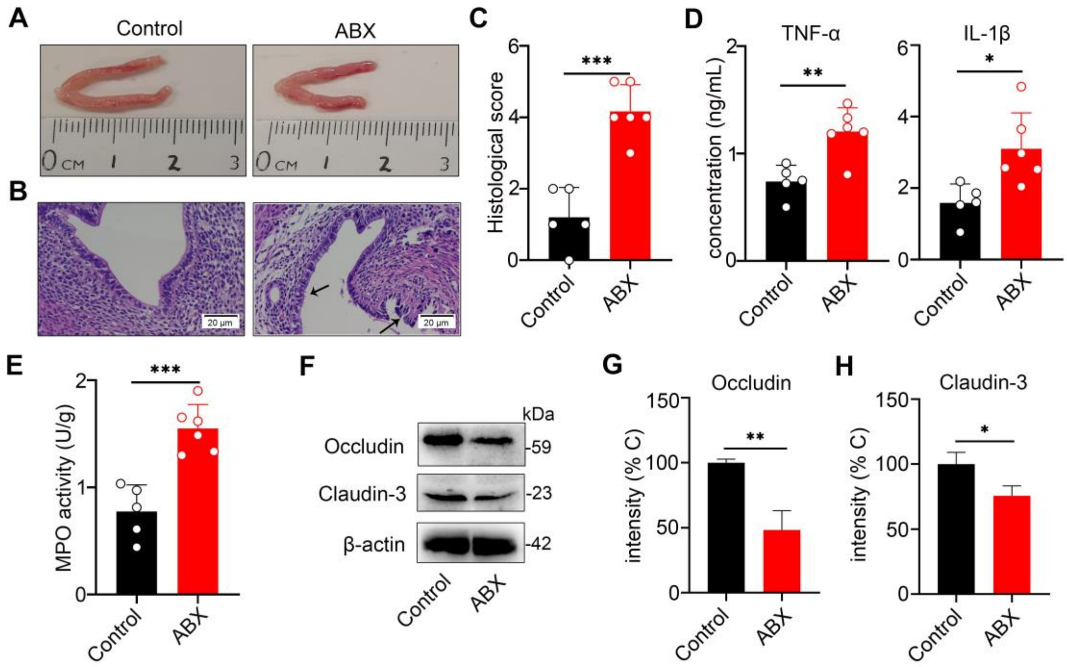

Fig. S2 Gut dysbiosis induces endometrial inflammation in mice. Mice were treated with ABX (1 g/L ampicillin, metronidazole and neomycin sulfate and 0.5 g/L vancomycin) for three weeks and uterine tissues were harvested. **A**. Representative macroscopic figures from different treatment groups are shown. **B.** Representative H&E-stained uterine sections are displayed. The black arrow shows endometrial damage (scale bar=20 μm). Histological score (**C**), TNF-α and IL-1β (**D**) and MPO activity (**E**) from indicated groups were determined (n=5-6). **F**. Levels of uterine occludin and claudin-3 protein from indicated groups were assessed by western blotting. **G-H**. Intensity analysis of occludin (**G**) and claudin-3 (**H**) was determined (n=5). Data are expressed as the mean ± SD and two-tailed Student's t test was performed (**C-E** and **G-H**). *$p < 0.05$, **$p < 0.01$ and ***$p < 0.001$ indicate significant differences. ABX, cocktail of antibiotics.

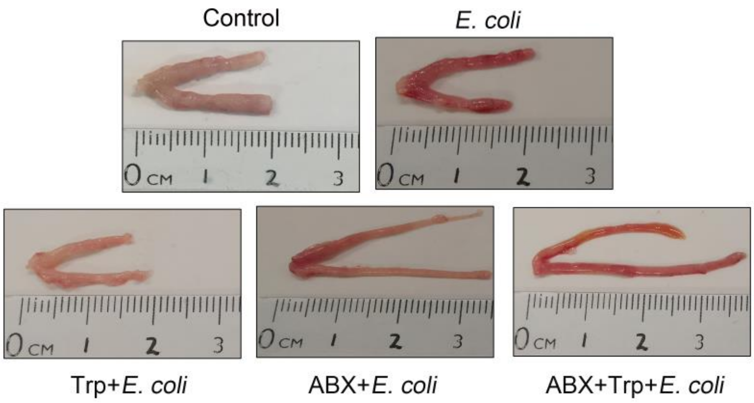

Fig. S3 Representative macroscopic images of Trp treated mice with or without ABX are shown.

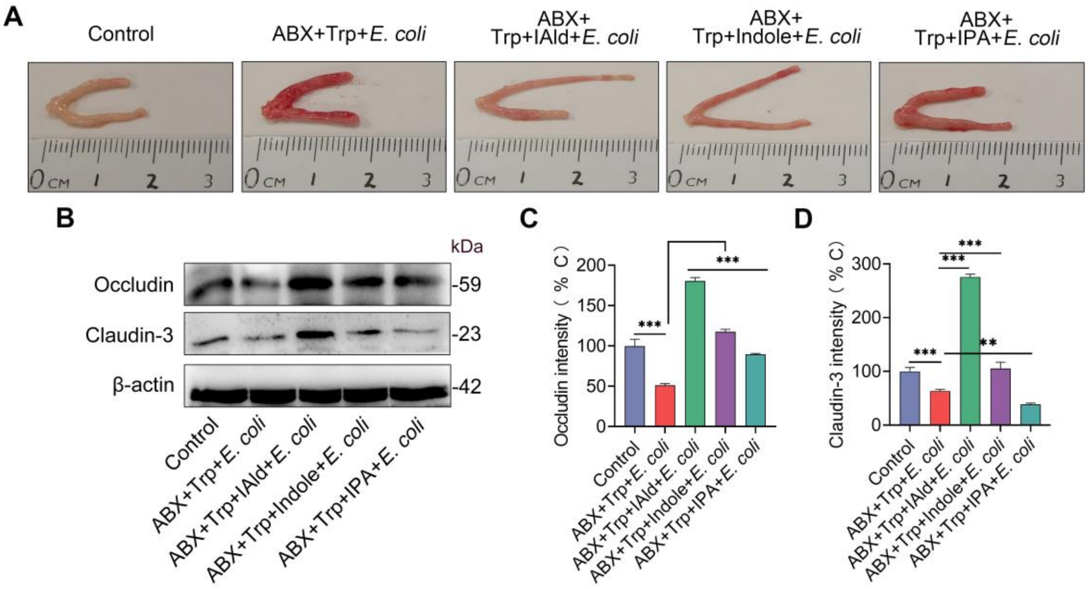

Fig. S4 IAld, Indole and IPA increase the expression of TJ proteins in *E. coli*-induced endometritis model. A. Representative macroscopic images from indicated mice are shown. **B**. Levels of uterine occludin and claudin-3 from indicated groups were assessed using western blotting. **C-D**. Intensity analysis of occludin (**C**) and claudin-3 (**D**) was performed based on western blotting (n=5). Data are expressed as the mean ± SD and one-way ANOVA (**C-D**) was performed. $**p < 0.01$ and $***p < 0.001$ indicate significant differences.

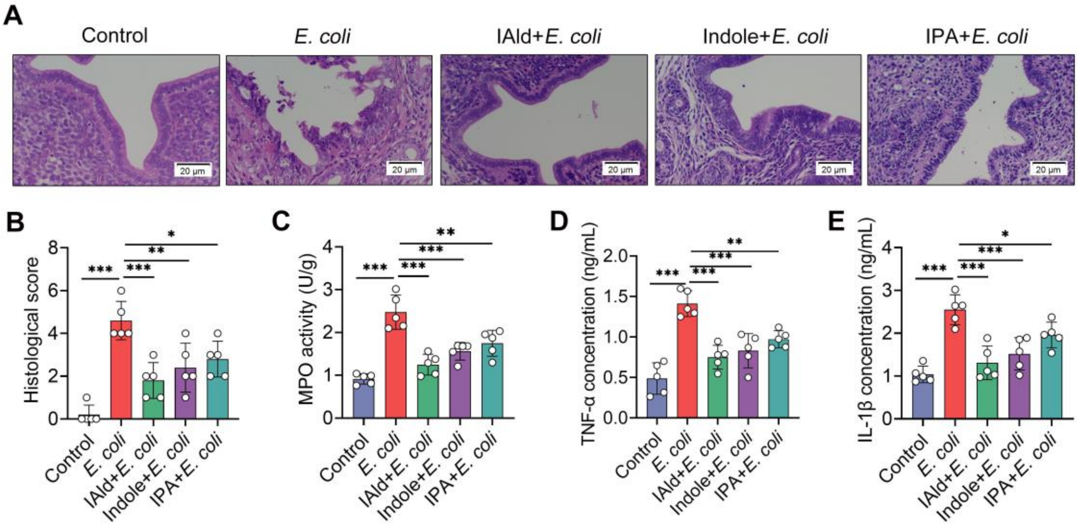

Fig. S5 IAld, Indole and IPA alleviate *E. coli*-induced endometritis in mice. A. Representative images of H&E-stained sections were shown in different treated groups (scale bar=20 μm). **B.** Histological scores were performed based on H&E staining (n=5). **C**-**E**. Inflammatory markers, including MPO activity (**C**), TNF-α (**D**) and IL-1β (**E**) were determined (n=5). Data are expressed as the mean ± SD and one-way ANOVA (**B**-**E**) was performed. *p < 0.05, **p < 0.01 and ***p < 0.001 indicate significant differences.

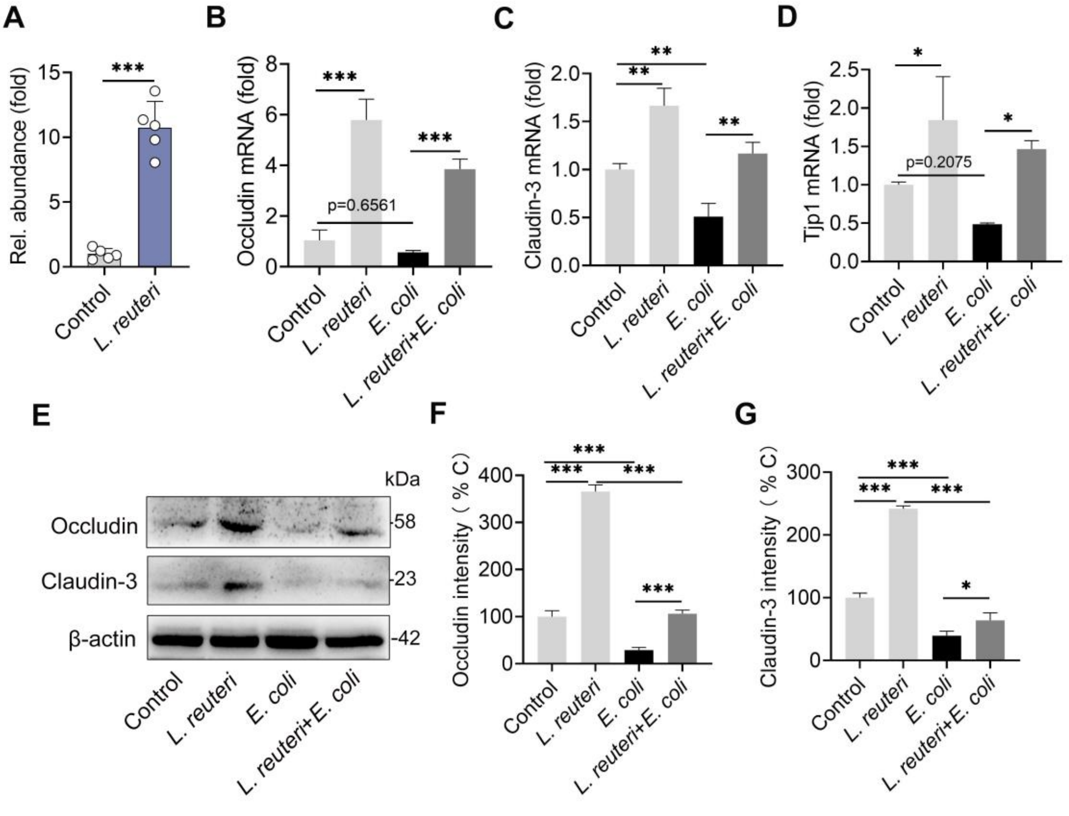

Fig. S6 ***L. reuteri*** **increases the expression of TJ in mouse uterine tissues.** Mice were treated with *L. reuteri* and *E. coli* and uterine tissues from indicated groups were assessed by qPCR or western blotting. **A**. Analysis of *L. reuteri* bacterial DNA isolated from fecal samples was performed in different treatment groups (n=5). The gene expression of *Occludin* (**B**), *Claudin-3* (**C**) and *Tjp1* (**D**) from different treated mice were determined using qPCR (n=5). **E.** Levels of occludin and claudin-3 from indicated groups were determined by western blotting. Intensity analysis of occludin (**F**) and claudin-3 (**G**) was performed (n=5). Data are expressed as the mean ± SD and two-tailed Student's t test (**A**) and one-way ANOVA (**B-D** and **F-G**) were performed. *p < 0.05, **p < 0.01 and ***p < 0.001 indicate significant differences.

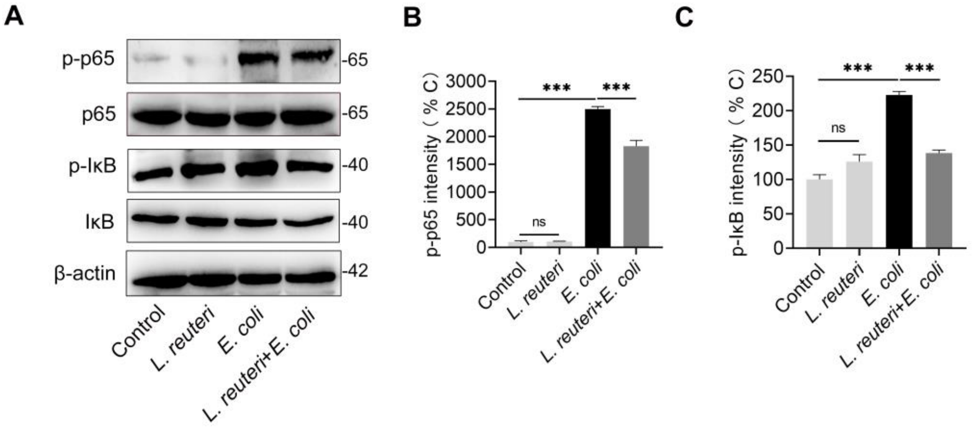

Fig. S7 ***L. reuteri*** **inhibits the activation of NF-κB caused by** ***E. coli*** **in uterine tissues. A.** The protein levels of the NF-κB pathway from indicated groups were assessed by western blotting. **B-C**. Intensity analysis of p-p65 (**B**) and p-κB (**C**) was performed (n=5). Data are expressed as the mean ± SD and one-way ANOVA (**B-C**) was performed. ***p < 0.001 indicates significant difference. ns, no significance.

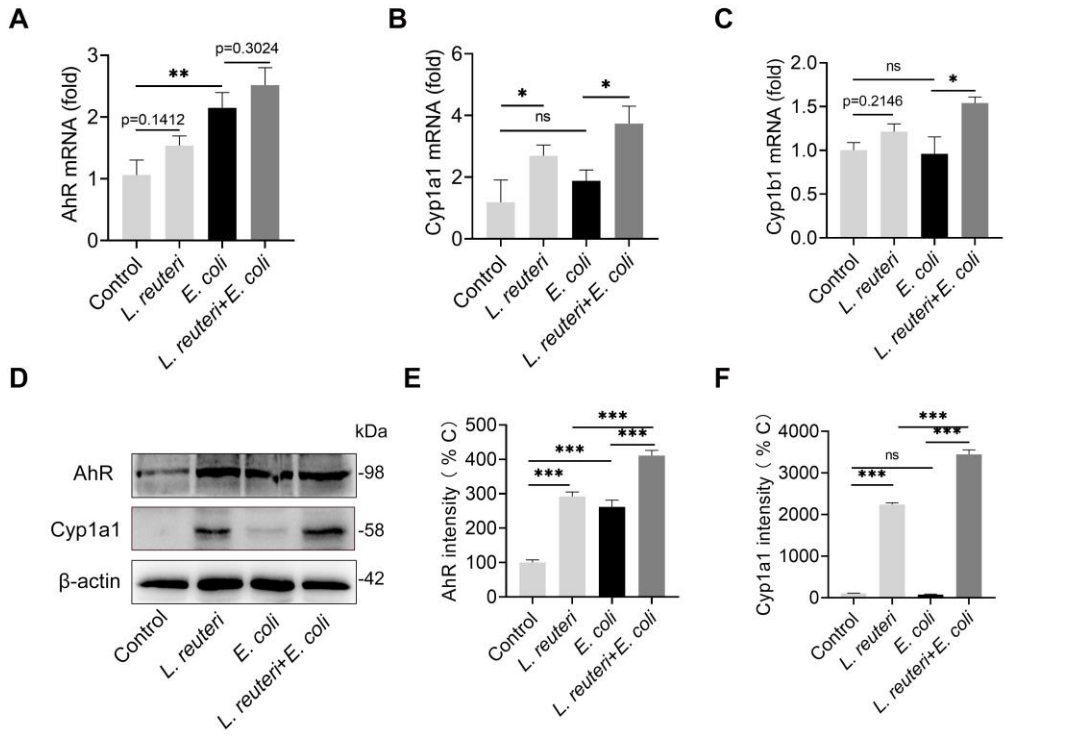

Fig. S8 *L. reuteri* activates the AhR pathway in uterine tissues. A-C. The mRNA levels of *AhR* (**A**), *Cyp1a1* (**B**) and *Cyp1b1* (**C**) from different treated groups were determined by qPCR (n=5). **D.** The protein levels of AhR and Cyp1a1 from different treated groups were assessed by western blotting. Intensive analysis of AhR (**E**) and Cyp1a1 (**F**) was performed (n=5). Data are expressed as the mean ± SD and one-way ANOVA (**A-C** and **E-F**) was performed. *$p < 0.05$, **$p < 0.01$ and ***$p < 0.001$ indicate significant differences. ns, no significance.

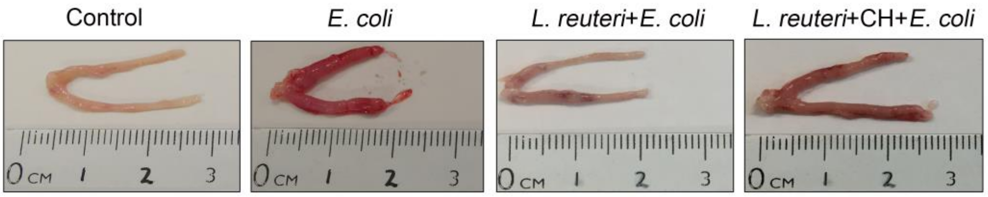

Fig. S9 Representative macroscopic images from *L. reuteri*-treated mice are shown.

Supplementary table S1. The oligonucleotides used in this study.

Gene	Primer	Sequence (5′-3′)
AhR	sense	5′-GAGCACAAATCAGAGACTGG-3′
	antisense	5′-TGGAGGAAGCATAGAAGACC-3′
Cyp1a1	sense	5′-CTCTTCCCTGGATGCCTTCAA-3′
	antisense	5′-GGATGTGGCCCTTCTCAAATG-3′

Cyp1b1	sense	5′-AATGAGGAGTTCGGGCGCACA-3′
	antisense	5′-GGCGTGTGGAATGGTGACAGG-3′
Occludin	sense	5′-CCCAGGCTTCTGGATCTATGT-3′
	antisense	5′-TCCATCTTTCTTCGGGTTTTCA-3′
Tjp1	sense	5′-GCCGCTAAGAGCACAGCAA-3′
	antisense	5′-TCCCCACTCTGAAAATGAGGA-3
Claudin-3	sense	5′-ACCAACTGCGTACAAGACGAG-3′
	antisense	5′-CAGAGCCGCCAACAGGAAA-3
L. reuteri	sense	5′-ACCGAGAACACCGCGTTATTT-3-3′
	antisense	5′-CATAACTTAACCAAACAATC AAAGATTGTCT -3
GAPDH	sense	5′-AACTTTGGCATTGTGGAAGG-3′
	antisense	5′-ACACATTGGGGGTAGGAACA-3′

Supplementary table S2. 16S rRNA sequences obtained in this study.

Sample name	Raw PE(#)	Raw Tags(#)	Clean Tags(#)
Control1	84,677	83,819	83,502
Control 2	87,427	86,463	86,048
Control 3	79,962	79,659	79,445
Control 4	89,218	87,186	86,817
Control 5	85,668	85,369	85,206
Control 6	80,209	79,590	79,346
ABX1	88,563	88,267	88,088
ABX2	85,803	85,530	85,337
ABX3	81,448	80,988	80,769
ABX4	87,682	86,879	86,620
ABX5	86,483	86,302	86,124
ABX6	79,283	79,227	79,104

Raw PE indicates original PE reads; Raw Tags are spliced sequences of Tags; Clean Tags are sequences of Raw Tags filtered out of low quality and short length.
